# Systematic Investigation into the Photoswitching and Thermal Properties of Arylazopyrazole-based MOF Host–Guest Complexes

**DOI:** 10.1021/acs.cgd.2c01384

**Published:** 2023-09-11

**Authors:** Kieran Griffiths, Jake L. Greenfield, Nathan R. Halcovitch, Matthew J. Fuchter, John M. Griffin

**Affiliations:** † Department of Chemistry, Lancaster University, Lancaster LA1 4YB, U.K.; ‡ Molecular Sciences Research Hub, Department of Chemistry, Imperial College London, London W12 0BZ, U.K.; § Center for Nanosystems Chemistry (CNC), Institut für Organische Chemie, Universität, Würzburg, Würzburg 97074, Germany

## Abstract

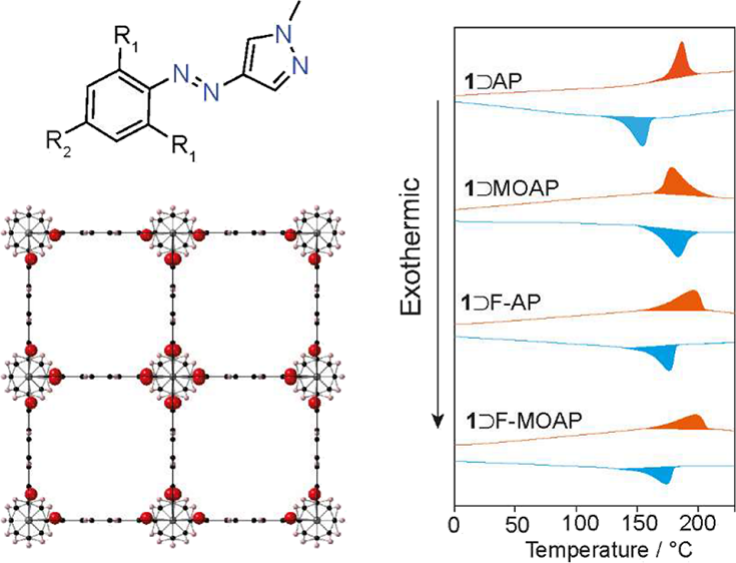

A series of arylazopyrazole-loaded metal–organic frameworks were synthesized with the general formula Zn_2_(BDC)_2_(DABCO)(AAP)_*x*_ (BDC = 1,4-benzenedicarboxylate; DABCO = 1,4-diazabicyclo-[2.2.2]octane; AAP = arylazopyrazole guest). The empty framework adopts a large pore tetragonal structure. Upon occlusion of the *E*-AAP guests, the frameworks contract to form narrow pore tetragonal structures. The extent of framework contraction is dependent on guest shapes and pendant groups and ranges between 1.5 and 5.8%. When irradiated with 365 nm light, the framework expands due to the photoisomerization of *E*-AAP to *Z*-AAP. The proportion of *Z*-isomer at the photostationary state varies between 19 and 57% for the AAP guests studied and appears to be limited by the framework which inhibits further isomerization once fully expanded. Interestingly, confinement within the framework significantly extends the thermal half-life of the *Z*-AAP isomers to a maximum of approximately 56 years. This finding provides scope for the design of photoresponsive host–guest complexes with high stability of the metastable isomer for long-duration information or energy storage applications.

## Abstract 2

A series of MOF host−guest complexes based on DMOF-1 containing arylazopyrazole guests was synthesized. The occluded guest molecules undergo reversible *E*-*Z* isomerization in response to UV light, which is accompanied by breathing of the MOF framework. The *Z* isomers show high thermal stability with half-lives of up to 56 years.

## 1 Introduction

Functional materials that respond to external stimuli are highly sought-after for a range of applications including sensing, optoelectronic devices, mechanical actuators, and information and energy storage.^1^ Light as a stimulus is very attractive as it can be used with high spatiotemporal control and produces no waste. Imparting photoresponsive properties on the molecular level usually requires the attachment of organic groups that can undergo suitable photochemistry. One particularly common approach to generate reversible light-responsive materials is the incorporation of molecular photoswitches that undergo reversible photoisomerization. Photoisomerization is often accompanied by changes in molecular geometry and organization and thus requires free space or flexibility around the photochromic unit; these spatial requirements can be difficult to engineer in solid systems.^2^ The most successful strategies to enable photoisomerization in solids are to either use soft materials like polymers or porous hosts such as metal–organic frameworks (MOFs).^3−5^ MOFs also offer the opportunity to examine structure–function relationships due to their well-defined periodic structures. However, while numerous photoswitches have been incorporated into MOFs, such as azobenzene derivatives,^6−9^ spiropyrans,^10−13^ and dithienylethenes,^14,15^ there are still no existing design strategies to achieve photoswitching in the solid state and the impact of the solid-state packing on the photoswitching properties remains largely unexplored.

Azobenzenes (ABs) are among the most highly studied photoswitches due to their attractive properties such as high cycling stability, large structural change, and high quantum yield.^16−20^ However, ABs can suffer from incomplete photoswitching due to the overlap in the absorption bands of the *E* and *Z* isomers. These issues can be addressed by specific functionalization to alter the absorption properties and thermal stability of the metastable *Z* isomer, for example, through *ortho*-functionalization.^21^ We recently showed that the photoswitching properties of ABs can also be significantly altered through confinement within MOF architectures. For azobenzene (AB) confined within the breathable MOF Zn_2_(BDC)_2_(DABCO) (**1,** where BDC = 1,4-benzenedicarboxylate and DABCO = 1,4-diazabicyclo-[2.2.2]octane), the half-life of the *Z* isomer produced through 365 nm irradiation was significantly increased to approximately 4.5 years, in comparison to 4 days in solution.^22^ However, the *Z* isomer population at the photostationary state (PSS) was reduced from approximately 77% in solution to 40% within the MOF. In contrast, the occlusion of AB into [Al(OH)(C_8_O_4_H_4_)] (Al-MIL-53) completely inhibits photoswitching due to the density of guest packing;^23^ however, loading with fluorinated ABs reduces the density of packing and allows photoisomerization to reach the solution-state PSS of up to 99%.^24^ Additionally, for 4-methoxyazobenzene occluded within **1**, a PSS of 99% of the *Z* isomer was obtained, but with only a slightly increased half-life of 6 days compared to 2 days in solution.^25^ The factors affecting the PSS and half-lives of occluded AB guests within MOFs are not well understood, although host–guest interactions and flexibility of the MOF are thought to play an important role.

**Figure 1:**
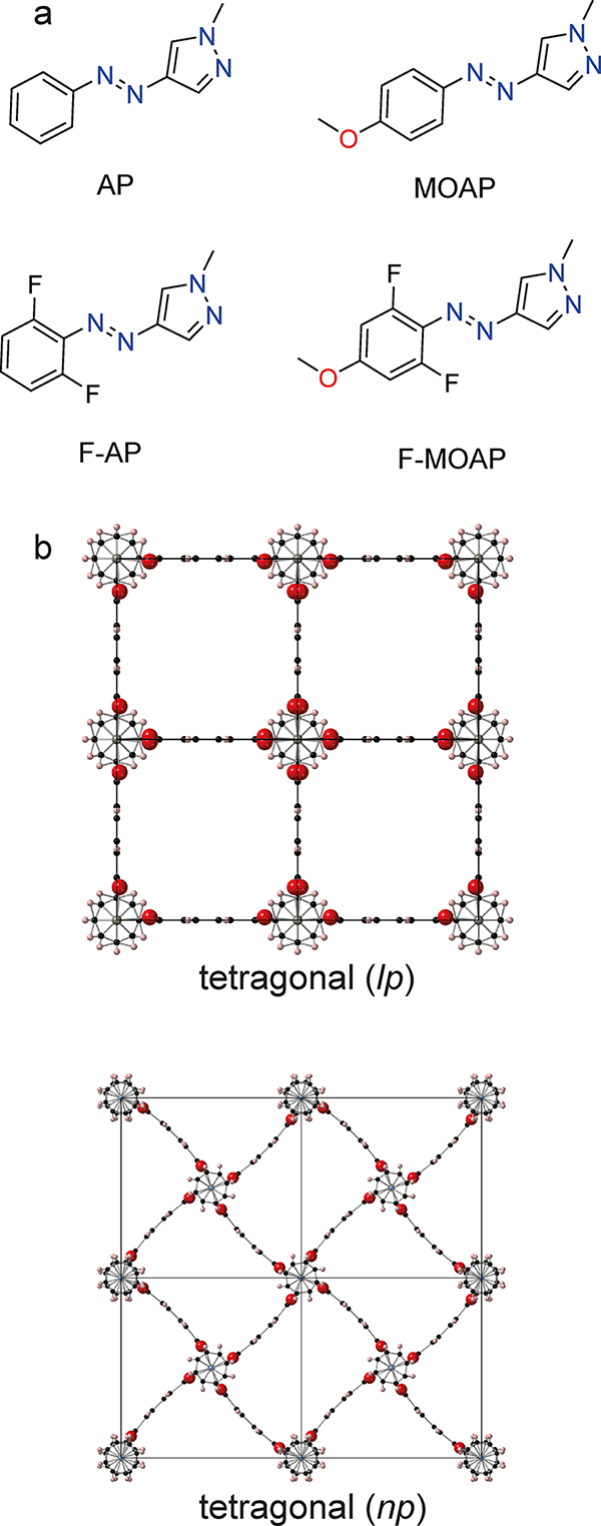
(a) AAP photoswitches used in this study and (b) tetragonal large pore (*lp*) and tetragonal narrow pore (*np*) structures of the MOF which are observed with occluded guest molecules. Carbon, oxygen, and hydrogen are represented by black, red, and white atoms, respectively.

Arylazopyrazoles (AAPs), a subset of the azo-based photoswitches, have been shown to offer an increased separation in the absorption bands of the *E* and *Z*-isomers as compared to ABs, as well as even higher thermal stability of the *Z*-isomers.^26^ For example, 1-methyl-4-(phenyldiazenyl-1H-pyrazole (AP, Figure 1) is highly addressable reaching a PSS of >98% *Z-*isomer under 355 nm irradiation and exhibiting a long thermal half-life of approximately 1000 days at room temperature in DMSO solution.^27^ Modification with a *para*-methoxy group (MOAP), also gives a PSS of 98% *Z*-MOAP under 365 nm irradiation with a half-life of 89 days.^28^*Ortho*-fluorination of AP (F-AP) gives a PSS of 92% *Z* isomer population under UVA irradiation.^29^ However, the half-life is increased to 46 years.

In this work, we present a systematic study of the photoswitching properties of a set of model AAPs confined within flexible MOF **1**. This represents the first time that AAPs have been incorporated into MOFs and studied. AAPs were chosen as they have similar overall size dimensions to ABs but display significantly different photophysical and photochemical properties. This includes an increased *E*/*Z* absorption band separation, which helps highlight differences between the effects of framework contraction and host–guest interactions on the PSS and half-life.

## 2 Experimental Section

All reagents and solvents were purchased from commercial suppliers, unless specified.

### 2.1 Synthesis of AAPs

AP was synthesized following reported a synthetic procedure (ESI).^27^ MOAP was synthesized following a reported synthetic procedure (ESI).^28^ F-AP was synthesized following a reported synthetic procedure (ESI).^29^ F-MOAP was synthesized with an adapted synthetic procedure (ESI).^28^

### 2.2 Synthesis of **1**

All reagents were obtained from Fluorochem and used without further purification. Zn_2_(BDC)_2_(DABCO) (**1**) was synthesized according to previously reported synthetic procedures.^30^ Zn(NO_3_)_2_·6H_2_O (0.5 g, 1.68 mmol) was sonicated in *N*,*N*-dimethylformamide (DMF, 20 mL) until fully dissolved; 1,4-dibenzendicarboxylic acid (0.28 g, 1.68 mmol) and DABCO (0.093 g, 0.84 mmol) were then added. The reactant solution was placed in a stainless-steel autoclave (Parr) with a Teflon lining with a 50 mL capacity. The solution was heated at 120 °C for 48 h and left to cool to room temperature. The colorless crystals were collected via vacuum filtration and washed with DMF (3 × 30 mL) before drying under ambient conditions to give an 81% yield based on Zn.

### 2.3 Loading of **1** with AAP

Samples of **1** were loaded with AAP using a previously published melt-infiltration procedure.^22,31,32^ As-prepared samples of **1** were first heated at 120 °C under vacuum for 24 h to remove DMF solvent molecules. The evacuated material (150 mg) was then mixed with a defined mass of *E*-AAP and heated at 160 °C for between 1 h. Excess AAP was removed by heating at 160 °C under vacuum for between 1 and 4 h, resulting in an 88–94% yield based on the removal of excess *E*-AAP. The duration of vacuum treatment was optimized such that the excess AAP was removed without removing occluded AAP from within the MOF structure.

### 2.4 Quantification of the Loading Level by UV–Vis Spectroscopy

UV–vis data were collected on a Cary 60 UV/vis spectrophotometer with a quartz cell (3 mL) within a 200–600 nm range. A calibration curve with known concentrations of *E*-AAP was constructed. AAP was extracted from **1**⊃AAP (50 mg) using benzene (10 mL × 4) and the filtrate was collected. The yellow filtrate was combined, and the solution was diluted to a volume of 50 mL.

### 2.5 Photostationary State Determination of Irradiated 1⊃AB_*x*_ Host–Guest Complexes

Twenty-five milligrams of **1**⊃AAP was suspended in benzene-*d*_6_ (0.5 mL) in an Eppendorf and shaken. The Eppendorf apron was centrifuged to separate the solid from the solution. A Bruker Avance III 400 NMR spectrometer with a 5 mm ^1^H-X broadband observe probe was used to collect ^1^H NMR data. The population ratio of *E*-AAP and *Z-*AAP isomers was determined from the integration of *E* and *Z* resonances in the ^1^H NMR spectrum based on literature values. The remaining solid was digested in DCl (1.5 mL) and DMSO-*d*_6_ (1.5 mL) and placed in a stainless-steel autoclave (Parr) with a Teflon lining with a 50 mL capacity. The suspension was heated for 12 h at 100 °C to yield a transparent solution. The ^1^H NMR spectrum of the solution was taken, and no residual *E*-AAP or *Z-*AAP resonances were detected. The process was repeated three separate times, and *Z*/*E* ratios were consistent.

### 2.6 UV Light Irradiation Procedure

Samples were irradiated with an OmniCure LX5 LED Head with a power of 425 mW and a 10 mm focusing lens. Fifty milligrams of finely ground **1**⊃AAP were spread homogeneously over a microscope slide. The powder was spread into a circle with a 1 cm radius which was approximately 0.5 mm thick so that irradiation was approximately uniform. The slide was placed under 365 nm light at a distance of 5 cm. The beam was set to 100% intensity and exposed for a fixed duration. The sample was incrementally agitated to allow all particulates to be exposed to the beam.

### 2.7 ^1^H and ^13^C Liquid State NMR

NMR spectra were recorded at 298 K using a Bruker AvanceIII HD Smart Probe 400 MHz spectrometer and automatically tuned and matched to the correct operating frequencies. TopSpin 3.5 and Mestrenova 8.0.0 S3 were used to apply the phase and baseline corrections. ^1^H and ^13^C NMR spectra were referenced to the residual solvent peak, and the ^19^F NMR spectra of organic molecules were referenced to hexafluorobenzene at −164.9 ppm and trifluoroacetic acid at −76.55 ppm. Signals are reported in terms of chemical shift (ppm) and coupling constants (Hz). Abbreviations for multiplicity are as follows: s, singlet; d, doublet; t, triplet; m, multiplet; br, broad; and hept, heptet.

### 2.8 Solid-State NMR

Solid-state NMR experiments were performed on a Bruker Avance III HD spectrometer operating at a magnetic field strength of 16.4 T, corresponding to ^1^H and ^13^C Larmor frequencies of 700 and 176 MHz, respectively. Spectra are referenced relative to tetramethylsilane (^13^C/^1^H) using the CH_3_ (^1^H = 1.1 ppm; ^13^C = 20.5 ppm) resonances of *L*-alanine as a secondary reference. ^13^C NMR spectra were recorded at a magic-angle spinning (MAS) rate of 16.0 kHz by using cross-polarization (CP) to transfer magnetization from ^1^H with a contact time of 3 ms. The CP pulse was ramped linearly from 70 to 100% power. ^1^H heteronuclear decoupling using two-pulse phase modulation (TPPM)^33^ with a pulse length of 4.8 μs and a radiofrequency field strength of 100 kHz was applied during acquisition. Spectra are the sum of 512 transients separated by a recycle interval of 10 s. The sample temperature in variable-temperature experiments was calibrated using Pb(NO_3_)_2_.^34^

### 2.9 X-Ray Powder Diffraction

X-ray powder diffraction (XRPD) patterns were measured with a Rigaku SmartLab X-ray diffractometer with a 9 kW rotating anode Cu-source equipped with a high-resolution Vertical θ/θ 4-Circle Goniometer and D/teX-ULTRA 250 High-Speed Position-Sensitive Detector System in reflectance mode. The system was configured with parallel-beam optics and a Ge(220) 2 bounce monochromator on the incident side. Powdered solid samples were prepared on glass slides. The measurements were performed as θ/2θ scans with a step size of 0.01 degrees. Variable temperature measurements were performed using an Anton-Parr BTS500 stage; the sample was loaded into a glass thin-walled capillary, and the stage was purged with nitrogen gas prior to measurement.

### 2.10 Density Functional Theory Calculations

First-principles calculations of NMR parameters were carried out under periodic boundary conditions using the CASTEP code^35^ employing the gauge-including projector augmented wave (GIPAW) algorithm.^36^ Prior to calculation of the NMR parameters, structures were fully geometry optimized, with all atomic positions to vary. For calculations on guest-free frameworks, the input atomic coordinates were taken from literature structures with the guest molecule atoms deleted.^30^ The structures were then optimized while the unit cell parameters were kept fixed to the experimental values. Atomic coordinates for optimized structures are given as cif files in the Supporting Information. Single-molecule calculations were carried out in a 20 × 20 × 20 Å cell with fixed cell parameters to ensure molecules remained isolated from periodic replicas. Full details are given in the Supporting Information (DFT Calculation Details).

## 3 Results and Discussion

### 3.1 Guest-Induced Behavior of AAP-MOF Host–Guest Complexes

**Figure 2:**
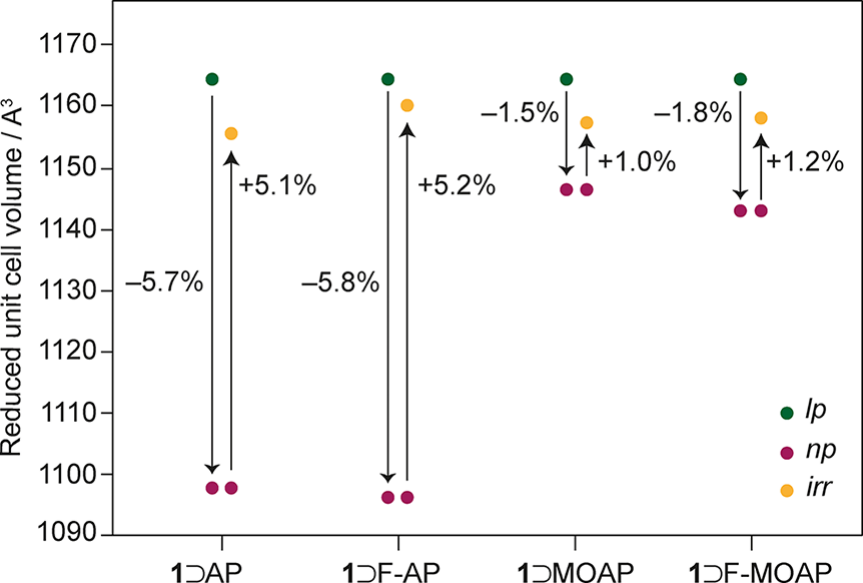
Scheme to display the relative contractions and expansions of the reduced unit cell of **1** in this study. *y*-axis error bars are within the diameters of the markers for each point. The differences between the points for each host–guest complex highlight the effects of guest occlusion causing a large-pore to narrow-pore contraction (*lp* → *np*) and irradiation with light causing a narrow-pore to irradiated structure expansion (*np* → *irr*).

To investigate the influence of MOF confinement on the photoswitching properties, AP, MOAP, F-AP, and F-MOAP were occluded by melt-infusion into **1** to ensure maximal loading of the guest molecules, and an excess guest was removed under heating and reduced pressure (S1–S2). Table 1 summarizes key crystallographic parameters for the complexes obtained from XRPD (Table S1) and profile fitting (Figures S3 and S4). The data for guest-free **1** are fully consistent with the previously reported large-pore (*lp*) tetragonal (*P*4/*mmm*) structure (Figures 1b and S4a). The AP-loaded MOFs all show a narrow pore (*np*) contracted unit cell characterized by a body-centered *I*4/*mcm* lattice that is known to result from the bending of the BDC linkers (Figures 1b and S4b–e). The 5.7–5.8% unit cell volume reduction observed for **1**⊃AP and **1**⊃F-AP is similar to previous work on **1**⊃AB, while for **1**⊃MOAP and **1**⊃F-MOAP, a smaller volume reduction of 1.5–1.8% is observed (Figure 2). The substitution pattern of the guest appears to influence the maximum loading level (Figure S5, TGA). For **1**⊃AP, the framework accommodates the equivalent of 1.25 molecules per pore, which is equivalent to maximum occupancy of the pores by ABs.^31,32^ A N_2_ gas sorption measurement gave a surface area of 2.18 m^2^ g^–1^, showing that the presence of AP guests in the pores results in a structure that is nonporous to the probe gas. *Ortho*-fluorination has little effect on the loading level (**1**⊃F-AP; 1.25 guests per pore). To a first approximation, fluorination should not significantly affect the overall length of the molecule, so similar loading levels could be expected purely by consideration of the molecular size with respect to the pore dimensions. Indeed, previous work^37^ has shown that similar maximum loading levels can be observed for azobenzenes fluorinated in different locations within **1**. In contrast, the addition of the bulkier *para*-methoxy groups decreases the loading level (**1**⊃MOAP, **1**⊃F-MOAP; 1.0 guest per pore). Interestingly, in comparison to the empty framework, the host–guest complexes with 1.25 guest molecules per pore show a greater unit cell contraction in comparison to those with 1.0 guest molecules per pore. The precise reason for this is not clear, but it is feasible that the higher occupancy of guests per pore gives a greater propensity for guest-linker interactions, which could drive the contraction of the framework. Indeed, it has been shown that relatively weak dispersion interactions can be important in controlling the contraction of flexible MOFs.^38^

**Table 1:** Selected Crystallographic Data for the **1**⊃AAP Compounds

compound	guest molecules per pore	space group	*a*/Å	*b*/Å	*c*/Å	α = β = γ/°	*V*/Å^3^	contraction (vol/%)
**1**		*P*4/*mmm*	10.98	10.98	9.65	90	1163.4	
**1**⊃AP	1.25	*I*4/*mcm*	15.06	15.06	19.35	90	4389.9	5.7
**1**⊃F-AP	1.25	*I*4/*mcm*	15.05	15.05	19.35	90	4383.0	5.8
**1**⊃MOAP	1.0	*I*4/*mcm*	15.41	15.41	19.31	90	4583.8	1.5
**1**⊃F-MOAP	1.0	*I*4/*mcm*	15.38	15.38	19.31	90	4570.5	1.8

**Figure 3:**
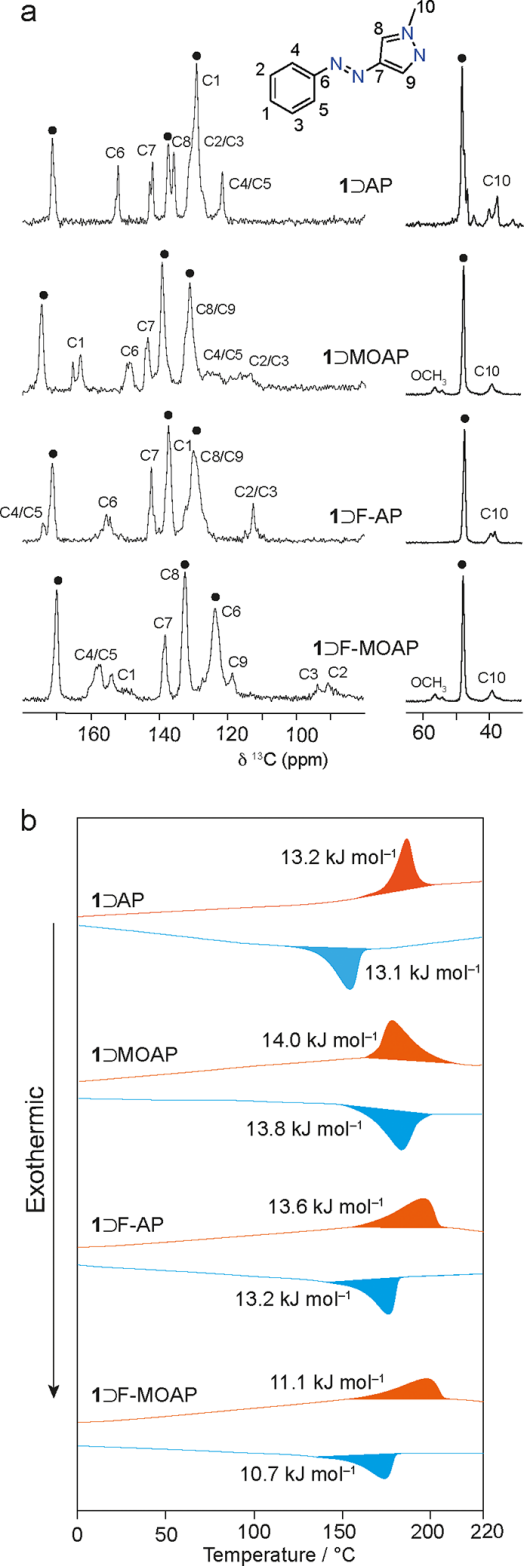
(a) ^13^C CPMAS NMR spectra of **1**⊃AAPs with a numbering scheme for the basic AAP unit. Black dots denote framework resonances. (b) First heating (orange) and cooling (blue) branches in DSC profiles for AAP-MOF complexes between 0 and 220 °C at 20 K min^–1^.

^13^C CPMAS NMR experiments were performed on the AAP-MOF complexes (Figure 3a). Each complex has distinct resonances corresponding to the crystallographically distinct BDC and DABCO carbons as well as the respective guest molecules. We have previously reported that the ^13^C chemical shifts of the BDC carbonyl and DABCO resonances are diagnostic of the nature of the contraction in the *np* structure.^22^ The framework resonances for the AAP-MOF complexes show noticeable shifts from the guest-free structure and lie within a narrow range, where DABCO resonances are between 47.5 and 48.3 ppm and carbonyl resonances of the BDC linker are between 171.1 and 171.5 ppm. Additionally, the carbonyl resonances have a distinct “shouldering” which was previously observed for **1**⊃AB. These shifts and lineshapes are consistent with the reported calculated values for a *np* tetragonal (*I*4/*mcm*) structure.^22^ Considering the guest molecules, it is apparent from the splitting of C10 and C11 resonances that they adopt multiple crystallographic environments within the pores of **1** (Figure 3a). Guest molecules occluded in **1**⊃AB and **1**⊃MOAB have been found to exhibit dynamics (pedal-motion of the central N=N linkage and/or ring flipping), which averages shifts for carbons on opposite sides of the benzene rings.^22^ DFT chemical shift calculations on isolated single molecules of AP and MOAP give poor agreement with the observed shifts in **1**⊃AP and **1**⊃MOAP, whereas models averaging shifts for different relative conformations of the five- and six-membered rings give good agreement with the experimental values (Table S2a–d). This suggests that AAP molecules also undergo rotational dynamics within the pores at a faster rate than the ^13^C chemical shift differences between different molecular conformations. Indeed, resonance broadening consistent with a reduction in the time scale of the AAP resonances is observed in spectra recorded at low temperatures (Figure S6).

The AAP-MOF host–guest complexes were studied using differential scanning calorimetry (DSC) to examine whether the *np* complexes could be thermally opened to an (open-pore) *op* structure which was previously observed for **1**⊃AB and **1**⊃MOAB.^22,32^ The *op* structure is equivalent to the *lp* structure albeit with a slight pore contraction (1.0–1.5%) around the confined guest molecule. Indeed, Figure 3b shows that the AAP-MOF complexes can reversibly transition between *np* and *op* structures, as evidenced by peaks with onset temperatures between 150 and 155 °C and can be cycled up to six times without any change in the phase transition enthalpies. The *np* ↔ *op* phase transition enthalpies (Table 2) are just over half that reported for **1**⊃AB (21.6 kJ mol^–1^),^22^ where the magnitude of the transition enthalpies was found to be linearly proportional to the amount of guest loaded. Considering that the AAP-MOF complexes are loaded to an equivalent or greater extent, this indicates weaker interactions between the host tetragonal *np* framework and the AAP guests. Interestingly, the magnitudes of the enthalpies for *np* ↔ *op* phase transitions are similar between complexes, even with considerable differences in unit cell contractions upon loading. The data in Table 2 show the entropy changes associated with the *np* ↔ *op* phase transitions. **1**⊃F-MOAP shows a slightly lower entropy change than the other complexes, which could suggest that F-MOAP is less ordered within the pores below the phase transition.

**Table 2:** Enthalpies, Δ*H*; Peak Temperatures, *T*_peak_, and Entropy Changes, Δ*S*, Associated with *np* ↔ *op* Phase Transitions for AAP-MOF Complexes Studied in This Work

	Δ*H /* kJ mol^–1^	*T*_peak_/K	Δ*S /* J K^–1^ mol^–1^
**1**⊃AP	13.2	438.3	30.1
**1**⊃F-AP	13.6	458.6	29.7
**1**⊃MOAP	14.0	434.4	32.2
**1**⊃F-MOAP	11.1	430.6	25.8

### 3.2 Irradiation of AAP-MOF Complexes

**Figure 4:**
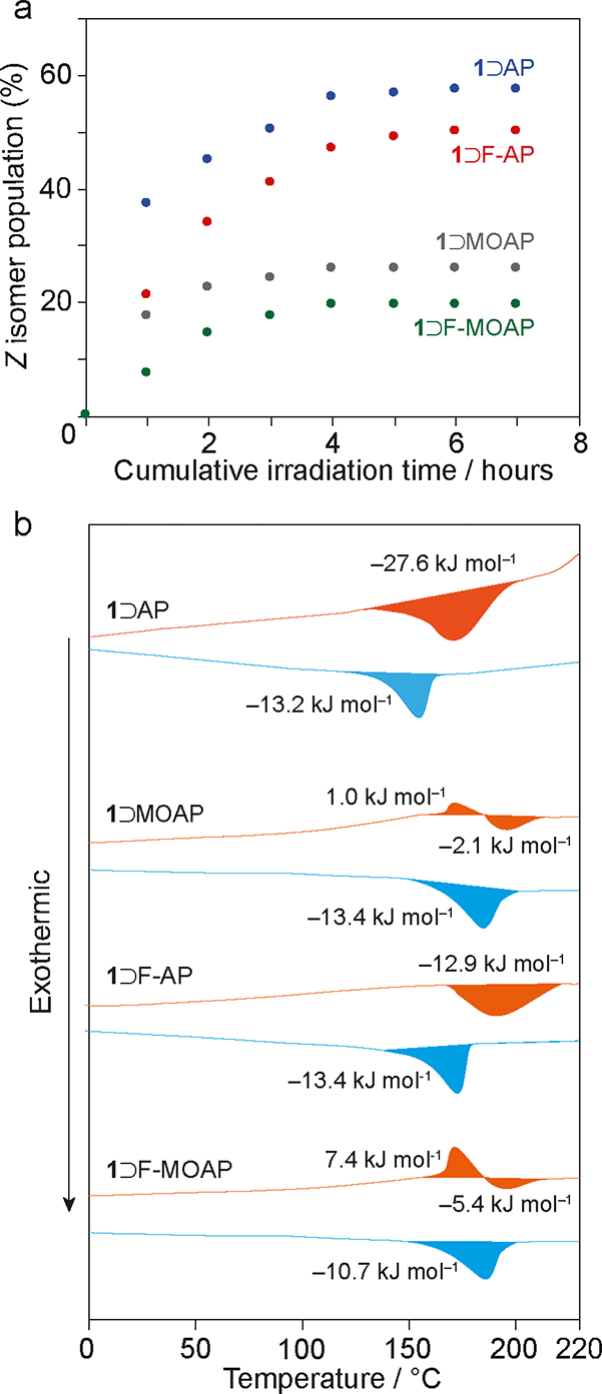
(a) *Z* isomer populations in AAP-MOF complexes as a function of irradiation time as measured by ^1^H NMR. (b) First heating (orange) and cooling (blue) branches of DSC experiments on irradiated AAP-MOF complexes between 0 and 220 °C at 20 K min^–1^.

Irradiation of the complexes in the solid state was performed using a 365 nm lamp, and subsequent solvent extraction of the guest AAP molecules followed by ^1^H NMR showed that photoisomerization to the *Z*-isomer occurs within all complexes (Figure S7). Figure 4a shows the proportion of *Z*-isomer measured by ^1^H NMR for irradiation times between 0 and 300 min. Under these conditions, each complex reached a PSS after approximately 4 h, which is slightly faster than **1**⊃AB (6 h) and **1**⊃MOAB (8 h). Regular agitation of the powder sample during irradiation and subsequent characterization (*vide infra*) confirmed that in each case, the PSS was not limited by light penetration effects. However, despite the AAPs demonstrating high *E* to *Z* switching efficiency in solution, when occluded in **1,** there is a large variation in the *Z* isomer population at the PSS. In **1**⊃AP and **1**⊃F-AP where the framework shows a large contraction, moderate *Z*-isomer populations of 57 and 50% were produced, respectively (Tables 3 and S3). However, for **1**⊃MOAP and in **1**⊃F-MOAP which show much smaller framework contractions, the conversion to the *Z*-isomer is modest at 26 and 19%, respectively. The observation of lower PSS values for **1**⊃MOAP and in **1**⊃F-MOAP is consistent with previous work on transition-metal-substituted analogues of **1**⊃AB showing that frameworks that are less contracted in the *np* structure give a lower conversion when irradiated.^31^ With these examples, the reported *Z* isomer populations at the PSS for AB and AAP molecules occluded in **1** range from 19 to 99%.^22,31,32^ The precise factors controlling the PSSs of MOF-photoswitch complexes remain unclear, but there is an apparent link with the degree of contraction in the unirradiated state. It is possible that structures that are less contracted in the unirradiated state indicate higher density packing arrangements of the guest molecules, resulting in less space for isomerization and, thereby, reduction of the PSS.

**Table 3:** Summary of Structural and Isomeric Changes Arising from the Irradiation of AAP-MOF Complexes

host–guest complex	*Z* isomer population (% at PSS)	unirradiated space group	volume Contraction (%)	irradiated space group
**1**⊃AP	57	*I*4/*mcm*	5.7	*P*4/*mmm*
**1**⊃F-AP	50	*I*4/*mcm*	5.8	*P*4/*mmm*
**1**⊃MOAP	26	*I*4/*mcm*	1.5	*P*4/*mmm*
**1**⊃F-MOAP	19	*I*4/*mcm*	1.8	*P*4/*mmm*
**1**⊃AB	40	*I*4/*mcm*	6.4	*P*4/*mmm*
**1**⊃MOAB	99	*Cmmm*	4.0	*P*4/*mmm*

Figure 4b shows DSC traces of the host–guest complexes after 300 min of irradiation. For irradiated **1**⊃AP and **1**⊃F-AP, a single exothermic transition is observed on the heating branch, whereas for **1**⊃MOAP and **1**⊃F-MOAP, small endothermic and exothermic features are observed. These features were observed previously for **1**⊃AB and **1**⊃MOAB, and experiments at different populations of the *Z*-isomer showed that this originates from either complete or partial canceling of the *np* → *op* endotherm by the exotherm due to the thermal reconversion from the *Z* to *E* isomer.^22,32^ DFT-calculated *E*/*Z* energy differences for the AAP guests studied here range between 40 and 55 kJ mol^–1^ (Table S4a–d). The net heat flow on the first heating branch corresponds well with the values calculated by taking into account the concurrent endothermic *np* → *op* phase transition of the framework and the exothermic *Z* → *E* conversion of the occluded AAP guests at the experimentally determined PSS (Table S5). On the first cooling, the exothermic features associated with the *op* → *np* phase transitions are identical to those of the nonirradiated samples. This confirms that the 0–220 °C thermal cycle causes full reconversion of the occluded *Z*-AAP to the *E*-AAP. Further heating/cooling cycles are the same as the nonirradiated forms, thus demonstrating the reversible nature of the irradiation-heating cycles.

XRPD measurements were performed to rationalize the PSS differences through structural changes that take place during UV irradiation. As the irradiation time increases, the reflections of the *np* tetragonal phase of the **1**⊃AAP complexes shift slightly and decrease in intensity and new reflections emerge. As the *Z*-isomer population increases, the relative intensities of the new reflections also increase, and after 240 min, they dominate the XRPD pattern. Indexing of each of the new phases for the **1**⊃AAP complexes reveals that the dominant phases are consistent with the tetragonal *lp* (*P*4/*mmm*) structure of **1** (Table 3, entries 1–4, Table S3, Figure S8a–d), albeit slightly contracted. Figure 2 shows the extent of expansion for the irradiated (*irr*) structures, which ranges from 1.0 to 5.2% relative to the *np* unit cell volumes. Across the series of host–guest complexes studied, the *irr* unit cell volumes are very similar (1154–1158 A^3^). Despite the unit cell expansions, a N_2_ gas sorption measurement on irradiated **1**⊃AP showed no significant increase in porosity (Table S6 and Figure S9), showing that the guest molecules still fill the space within the pores. In conjunction with previous data for **1**⊃AB (Table 3, entry 5), this strongly supports that the structural change is a global transformation, which incorporates both *E* and *Z* isomers, rather than segregation into *E*-isomer and *Z*-isomer-containing domains. Interestingly, even with starkly contrasting *E*/*Z* proportions, the irradiated **1**⊃AB and **1**⊃AAP complex XRPD patterns are all dominated by the *lp* phase at the PSS. This suggests that further photoisomerization from the *E* to the *Z* isomer is inhibited once the structure has globally converted to the *lp* form. This supports the proposed explanation for the lower *Z*-isomer proportions of **1**⊃MOAP and **1**⊃F-MOAP at the PSS, where the guest-induced contraction in the *np* form is smaller, and therefore, there is less volume for framework expansion to subsequently allow isomerization (Table 3, entries 2–4). However, the inhibition of *E* to *Z* isomerization by the *lp* structure is limited to those complexes that adopt the tetragonal *np* structure in the unirradiated state (Table 3, entries 1–5). For **1**⊃MOAB (Table 3, entry 6), which adopts the orthorhombic *np* structure in the unirradiated state, the *E* to *Z* photoisomerization continues beyond reaching the *lp* tetragonal structure. This points toward an intrinsic difference in the guest-induced symmetry and its effect on the photoswitching properties of the resultant complexes.

**Figure 5:**
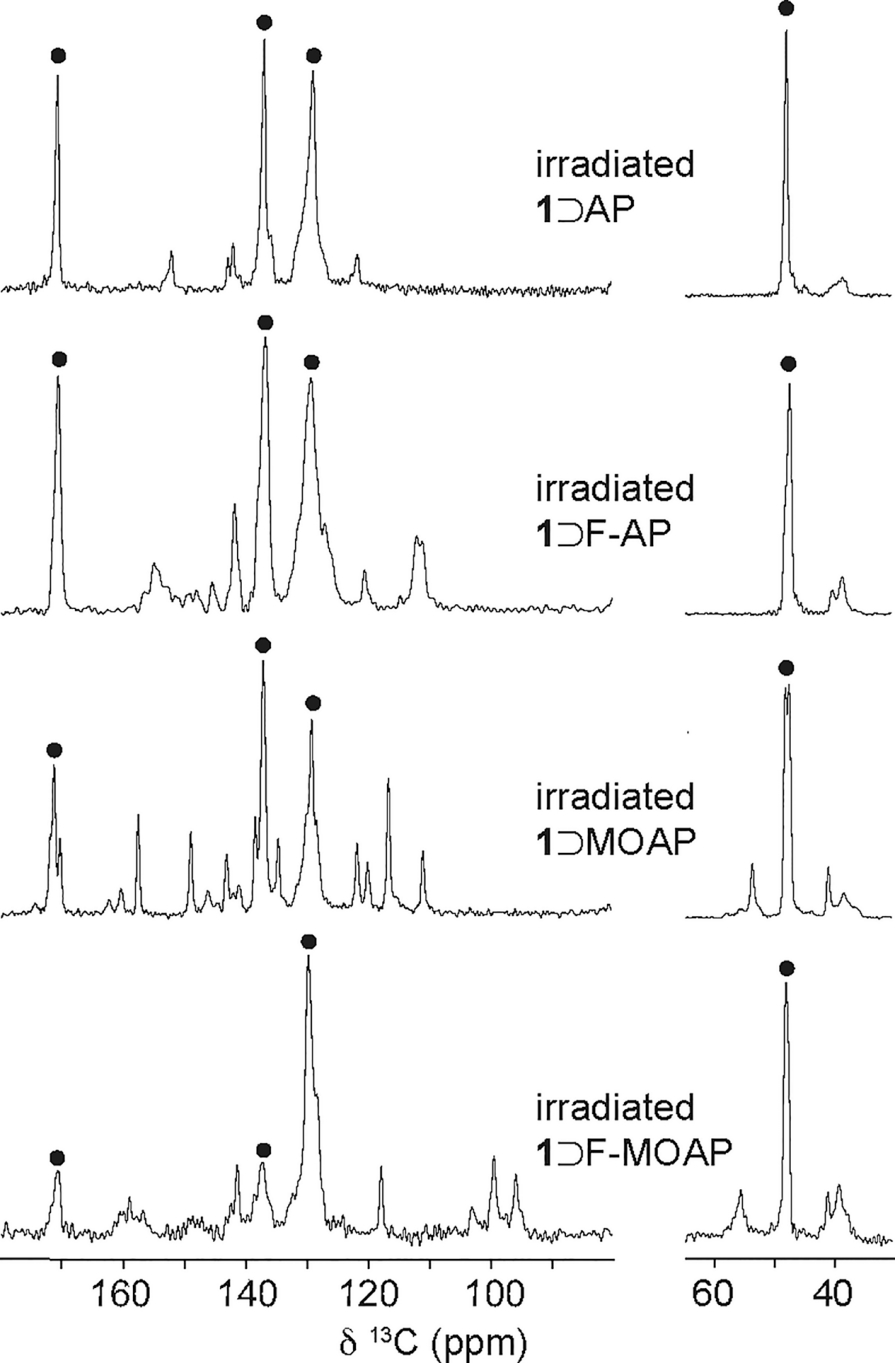
^13^C CPMAS NMR spectra of irradiated AAP-MOF complexes recorded at ambient temperature (black dots denote framework resonances).

Figure 5 shows the ^13^C CPMAS NMR spectra of the irradiated AAP-MOF complexes. For **1**⊃AP, the three distinct DABCO environments consolidate to a single resonance at 47.9 ppm, and the carbonyl peak loses the distinct “shouldering” and shifts to 171.1 ppm. This consolidation is similar for **1**⊃F-AP and both of these factors are consistent with an increase in symmetry and the framework expanding from the *np* tetragonal structure to the *op* tetragonal structure. For **1**⊃AP and **1**⊃F-AP at room temperature, no resonances of *Z*-AP are observed, and resonances of *E*-AP are of lower intensity than the nonirradiated form. However, for **1**⊃MOAP and **1**⊃F-MOAP, resonances for both the *E*-isomer and *Z*-isomers are evident. When the temperature is reduced to 220 K, three additional resonances emerge for **1**⊃AP at 157.9, 144.6, and 121.1 ppm (Figure S6). For the *Z*-AAP isomers, simulations whereby shifts for chemically equivalent species are averaged show good agreement with the experimental data, suggesting that the *Z*-AAP molecules are undergoing rapid rotational motion within the pores (Table S7a–d). The absence of *Z*-AP and *Z*-F-AP resonances at ambient temperature suggests that the time scale of the motion at this temperature broadens the resonances beyond detection. The behavior of the **1**⊃AP and **1**⊃F-AP system is similar overall to **1**⊃AB, where the nonlinear geometry of the Z-isomers facilitates molecular tumbling in the pores. As there is a distinct difference between the time-scale of the motion for significantly contracted structures (**1**⊃AP and **1**⊃F-AP; 1.25 molecules per pore) and slightly contracted pores (**1**⊃MOAP and **1**⊃F-MOAP; 1 molecule per pore), the ordering and packing of guest molecules in the unirradiated framework must have a considerable influence free volume available to the *Z* isomers in the irradiated complexes.

### 3.3 Stability and Thermal Reconversion of AAP-MOF Complexes

Through the occlusion of AAP photoswitches into the framework of **1**, it is possible to control the opening and closing of the pore structure through irradiation with both light and temperature. Irradiation (365 nm) of **1**⊃AAP complexes converts ground-state *E*-AAP molecules to the metastable *Z*-AAP isomer, whereupon the flexible structure of **1** opens to accommodate the shape-change (*np* → *op*). Heating irradiated **1**⊃AAP above the endothermic *np* → *op* threshold temperature causes thermal reconversion of *Z*-AAP guest molecules to the *E*-AAP isomer, and upon cooling, the **1** framework closes around the guest via an *op* → *np* phase transition. The exotherm associated with the thermally driven reconversion of *Z*-AAP to *E*-AAP during heating is either comparable to or greater in magnitude to the *np* → *op* endotherm, meaning that net heat flow during the heating branch is either negligible or exothermic. Additionally, upon cooling, an *op* → *np* exotherm is also observed. This means that over one full heating and cooling cycle of the irradiated complexes, there are net energy outputs of 40.8 kJ mol^–1^ for **1**⊃AP, 26.3 kJ mol^–1^ for **1**⊃F-AP, 16.5 kJ mol^–1^ for **1**⊃MOAP, and 8.7 kJ mol^–1^ for **1**⊃F-MOAP. These values are modest in comparison to previously studied **1**⊃AB complexes (up to 86 kJ mol^–1^), but they demonstrate the capability of these complexes to store energy within the metastable *Z* isomers of the AAP guest molecules.

**Figure 6:**
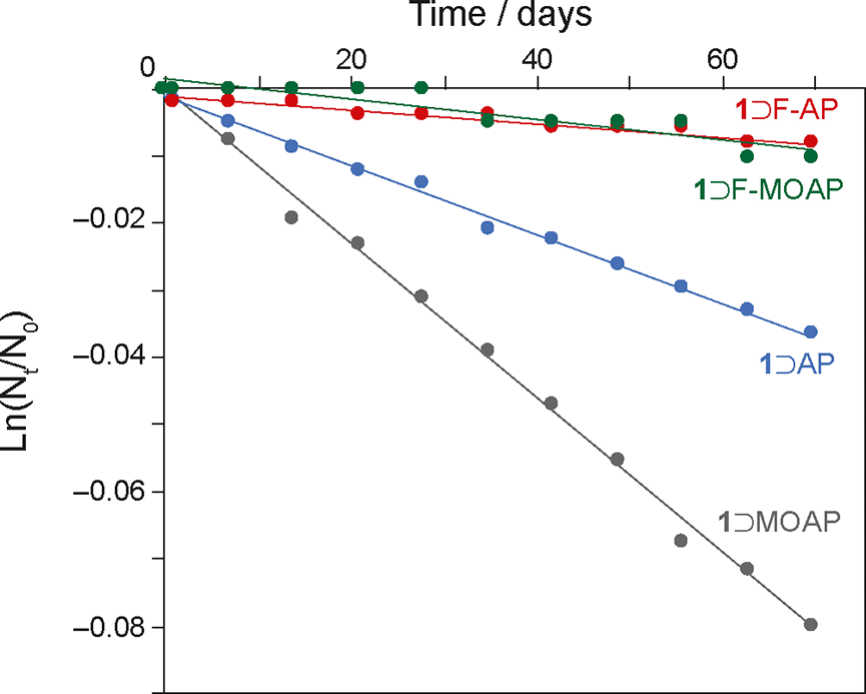
Logarithmic plot of the normalized population of *Z*-AAP occluded within irradiated **1**⊃AAP complexes at ambient temperature in the dark as a function of time.

A key property of any photoswitch is the rate of spontaneous thermal reconversion of the metastable isomer to the ground-state isomer at ambient temperature in the dark. In general, this is expected to occur via first-order Arrhenius kinetics, such that the Z-isomer stability can be parametrized by a single rate constant or half-life. For many applications, it is desirable for the metastable isomer to exhibit a long half-life so that photochemical control can be employed without spontaneous changes in the proportions of *E*/*Z* isomers. To estimate the half-lives for the AAP-MOF complexes, irradiated samples were kept in the dark for 3 months at ambient temperature, with portions extracted and the *Z*-isomer quantified via ^1^H NMR at regular intervals (Table S8a–d). Figure 6 confirms that *Z* → *E* thermal reconversion at ambient temperature follows first order Arrhenius kinetics for each of the complexes. Linear fits to the data show all AAPs studied exhibit significantly longer *Z*-isomer half-lives when occluded in **1**, as compared to those in solution (see Table 4). We note that for F-AP which already shows a very long half-life of 46 years in solution, there is considerable uncertainty in the half-life reported here due to the very small reduction in *Z* isomer population over the 3 month period, and the true half-life could be even longer. Previously reported AB and MOAB also show significant increases in half-life upon occlusion within **1**, so it seems likely that occlusion of other azo-photoswitches will also lengthen the half-life of the *Z*-isomer. The measured half-lives of MOF-AAPs do not correlate with the volume contraction of unirradiated and although it is increased by the confinement, the thermal stability still seems to be dependent on a complex interplay between multiple factors, presumably including the intrinsic molecular stability as well as host guest interactions, contraction/confinement within the pore, and any dynamics that may be present.

**Table 4:** Half-Lives of AAP and AB Guest Molecules in Solution and within Complexes of **1.**

guest	half-life of *Z* isomer (years)^a^	half-life of *Z* isomer in **1** (years)	change in half-life (%)
AP	2.7	3.8	140
F-AP	46.0	∼56	122
MOAP	0.2	1.7	850
F-MOAP	not determined	∼21	N.A.
AB	1.1 × 10^–2^	4.5^b^	380
MOAB	4.3 × 10^–3^	2.1 × 10^–2^^c^	490

a Half-life in DMSO.^27−29^

b Taken from ref (22).

c Taken from ref (32).

## 4 Conclusions

When azopyrazole photoswitches are occluded within the flexible metal organic framework **1**, a guest-induced framework contraction ranging between 1.5 and 5.8% is observed, resulting in a *np* tetragonal structure. All complexes show reversible thermally driven phase transitions between the *np* and *op* structures in the range 150–155 °C. Solid-state ^13^C CPMAS NMR confirms the dynamic motion of the photoswitches while occluded in the MOF framework. The degree of contraction appears to be correlated with the photostationary state of the *Z*-isomer under 365 nm irradiation, although it is likely that the ordering of guest molecules within the pores and host–guest interactions also influence the photostationary state. Irradiation causes the framework to expand to the *lp* form to accommodate the structural rearrangement of the occluded *Z*-isomer photoswitches, but once the framework is in the *lp* form, the isomerization can no longer take place. This leads to generally reduced photostationary states for the AAP photoswitches as compared to in free solution. However, the thermal half-lives of the *Z*-isomers of the occluded guests are all increased in the complexes. The increase in half-life ranges from 122 to 850% as compared to the solution state. This work demonstrates the importance of the framework flexibility in the design of confined photoswitch systems and demonstrates that lengthening of the *Z*-isomer half-life is a likely consequence of confinement in a flexible framework.

## Supporting Information Available

Full synthetic details; DFT calculation details; additional DSC data; optical microscopy; XRPD data and Le Bail fits; crystallographic data; TGA data; and gas sorption analysis (PDF)
