# Supplementary material for: Systematic Investigation into the Photoswitching and Thermal Properties of Arylazopyrazole-based MOF Host–Guest Complexes
Source: Cryst Growth Des. 2023 Sep 11;23(10):7044–52. doi: 10.1021/acs.cgd.2c01384 (PMC10557064; doi:10.1021/acs.cgd.2c01384)
Supplement: Supplementary file 1 — cg2c01384_si_001.pdf [file cg2c01384_si_001.pdf]

Supporting Information for

A Systematic Investigation into the Photoswitching and Thermal Properties of Arylazopyrazole-based MOF Host-Guest Complexes

Kieran Griffiths[1], Jake L. Greenfield[2,3], Nathan R. Halcovitch[1], Matthew J. Fuchter[2], John M. Griffin[1]*

[1]Department of Chemistry, Lancaster University, Lancaster LA1 4YB, UK

[2]Molecular Sciences Research Hub, Department of Chemistry, Imperial College London, London W12 0BZ, UK

[3]Center for Nanosystems Chemistry (CNC), Institut für Organische Chemie, Universität, Würzburg, Würzburg 97074, Germany

*Corresponding author email: j.griffin@lancaster.ac.uk

Synthesis of F-MOAP

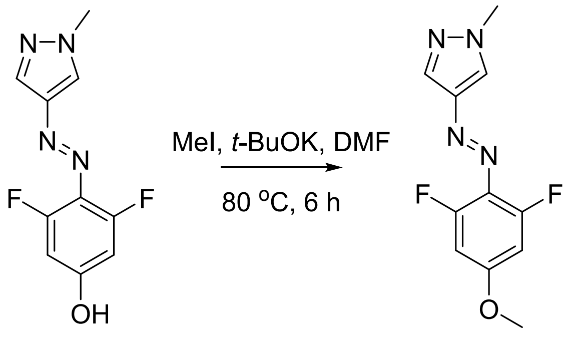

S1 **FMO4PzH**

F-AP (200 mg, 0.8 mmol, 1 equiv), *t*-BuOK (122 mg, 1.1 mmol, 1.3 equiv), and CH_3I (0.26 mL, 4.2 mmol, 5 equiv) in 10 mL of anhydrous DMF was stirred at 80 °C for 6 h. The reaction mixture was quenched by adding water. A solid precipitated out over time and collected via filtration and washing with water. The filter cake was dissolved in EtOAc and washed with LiCl (5 wt% in water, 2 × 20 mL). The organic phase was dried over $MgSO_4$ and concentrated under reduced pressure. Purification by column chromatography (EtOAc:Hexane from 0:1 to 1:1) afforded **F-AP** as a yellow solid (140 mg, 66%); ^{1}H NMR (400 MHz, 298 K, DMSO-d_6): δ 8.47 (s, 1H), 7.93 (s, 1H), 6.95-6.89 (m, 2H), 3.91 (s, 3H), 3.85 (s, 3H) ppm; ^{13}C NMR (100 MHz, 298 K, DMSO-d_6): δ 160.6, 154.9, 150.1, 141.9, 132.0, 128.2, 99.8, 56.5, 39.7 ppm; ^{19}F{^{1}H} NMR (378 MHz, 298 K, DMSO-d_6): δ −122.07 ppm; HRMS (APCI, MeCN): *m/z* calcd: 253.0901; found 253.0899 (Error -0.8 ppm).

DFT Calculation Details

First-principles calculations of NMR parameters were carried out under periodic boundary conditions using the CASTEP code[1] employing the gauge-including projector augmented wave (GIPAW) algorithm,[2] which allows the reconstruction of the all-electron wave function in the presence of a magnetic field. The CASTEP calculations employed the generalised gradient approximation Perdew–Burke–Ernzerhof exchange-correlation functional,[3] and core–valence interactions were described by ultrasoft pseudopotentials.[4] Single-molecule calculations were

carried out in a 20 × 20 × 20 Å cell with fixed cell parameters to ensure molecules remained isolated from periodic replicas. Geometry optimisations and NMR calculations were carried out using a planewave energy cut-off of 60 Ry, and for crystal structures, a k-point spacing of 0.05 2π Å^{-1} was used. For single-molecule calculations, a single k-point at the fractional coordinate (0.25, 0.25, 0.25) was used. The calculations generate the absolute shielding tensor (σ) in the crystal frame. Diagonalisation of the symmetric part of σ yields the three principal components, σ_{XX}, σ_{YY}, and σ_{ZZ}. The isotropic shielding, σ_{iso}, is given by (1/3) Tr [σ]. The isotropic chemical shift, δ_{iso}, is given by $\sigma_{ref} - \sigma_{iso}$, where σ_{ref} is a reference shielding. Reference shieldings were determined by comparison of experimental chemical shifts for *L*-alanine with shieldings obtained from a calculation on a fully optimised crystal structure[7] (Cambridge Structural Database code LALNIN22). For ^{1}H and ^{13}C, reference shieldings were determined from the *y* intercept of a linear fit to the experimental shifts versus calculated shielding, with the gradient of the fit fixed to −1. Calculated shieldings for the three methyl protons were averaged to account for rapid rotation of the methyl group. Respective reference shieldings of 30.2 and 168.4 ppm were obtained for ^{1}H and ^{13}C. Calculated chemical shifts for the individual carbons in DABCO groups were averaged to account for the fast rotational dynamics of this group.

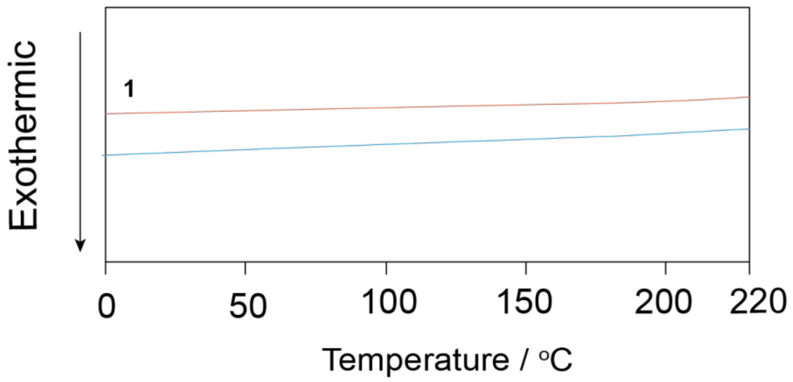

Figure S1a. DSC traces for guest-free **1.** No thermal features are observed between 0 - 220 °C on heating (orange), or cooling (blue).

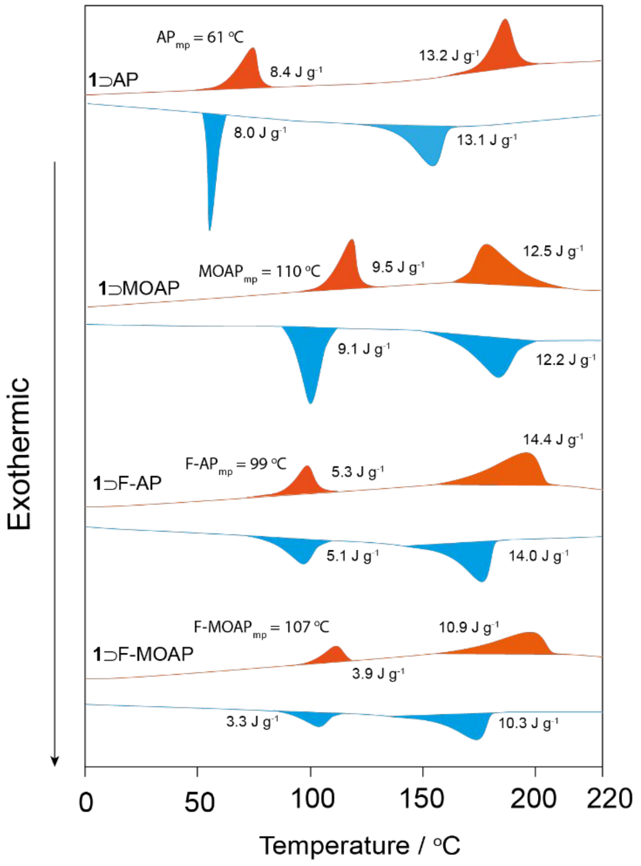

Figure S1b. DSC traces for synthesised **1**⊃AP, **1**⊃F-AP, **1**⊃MOAP, and **1**⊃F-MOAP prior to vacuum treatment, heating (orange), cooling (blue). Residual melting and crystallisation transitions are observed for excess guest molecule at the expected temperatures. Subsequent vacuum treatment removes the excess (Figure 3).

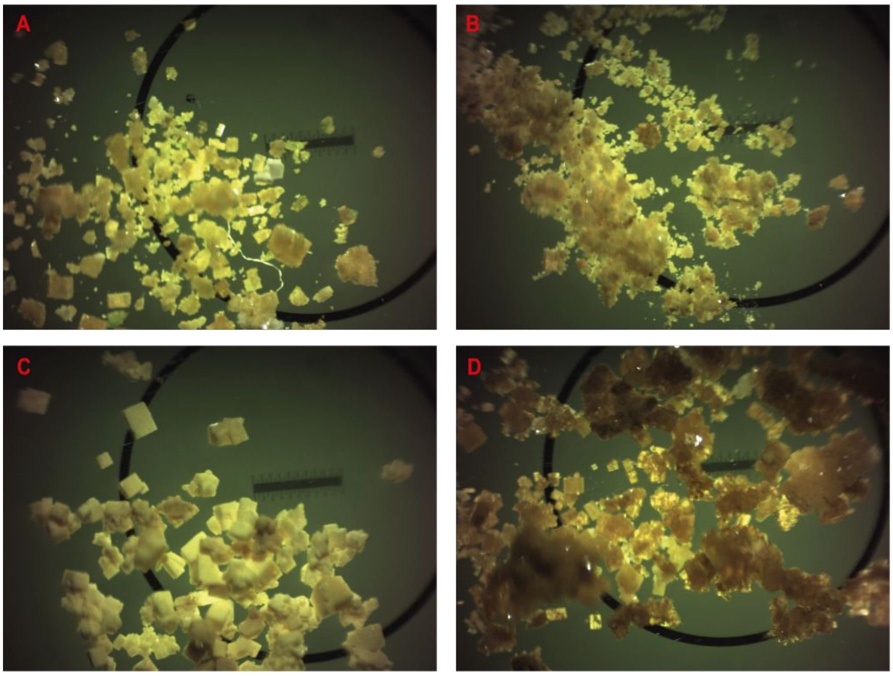

Figure S2. Optical microscopy photographs of **A**) **1**⊃AP, **B**) **1**⊃F-AP, **C**) **1**⊃MOAP, and **D**) **1**⊃F-MOAP; the black scale bar near the centres of the images represents 1 mm with individual graduations at 0.01 mm.

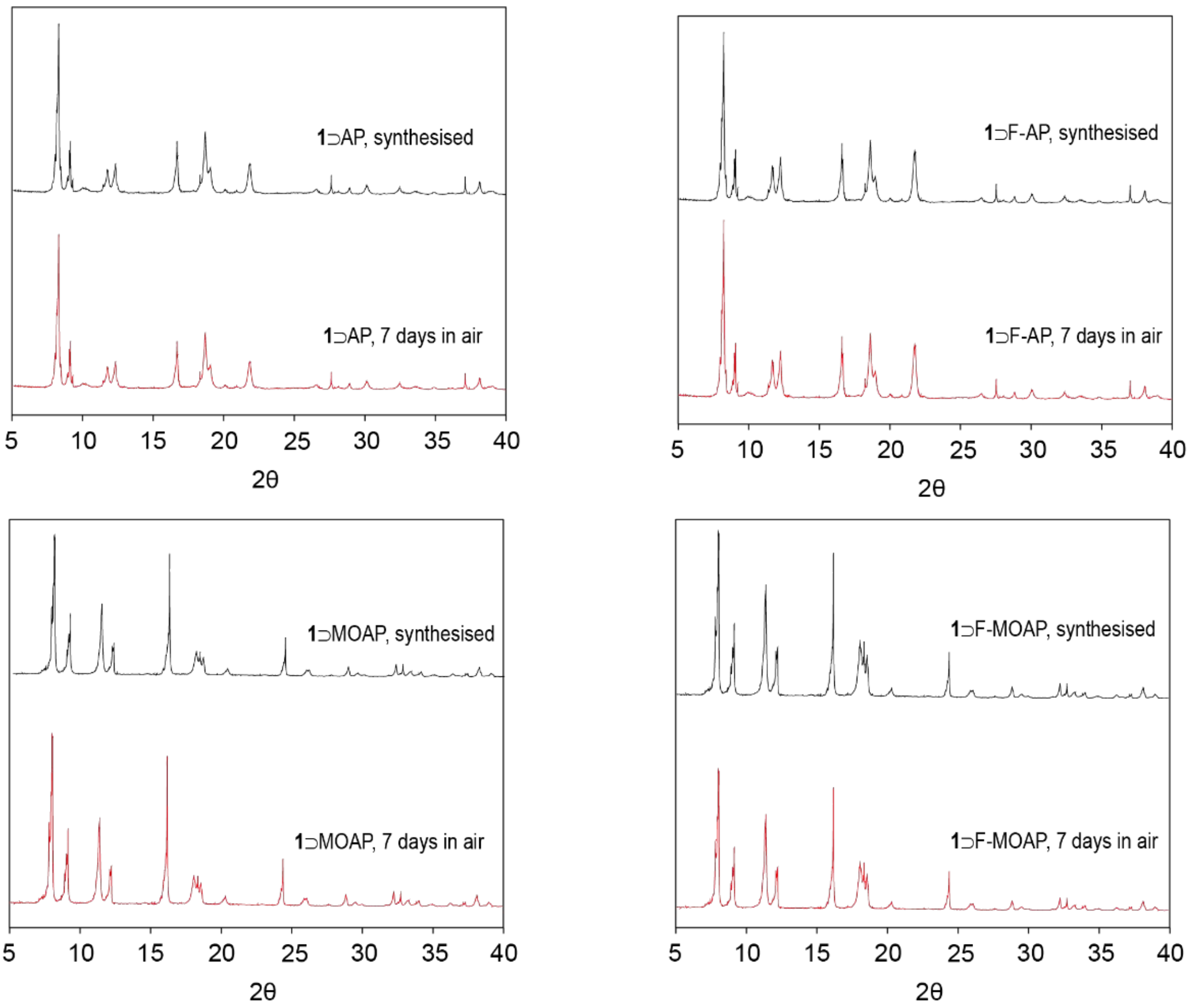

Figure S3. XRPD profiles for synthesised **1**⊃AP, **1**⊃F-AP, **1**⊃MOAP, and **1**⊃F-MOAP. Through the occlusion of guest molecules, the framework structure remains stable in air and no changes are seen in the XRPD pattern over a period of 1 week.

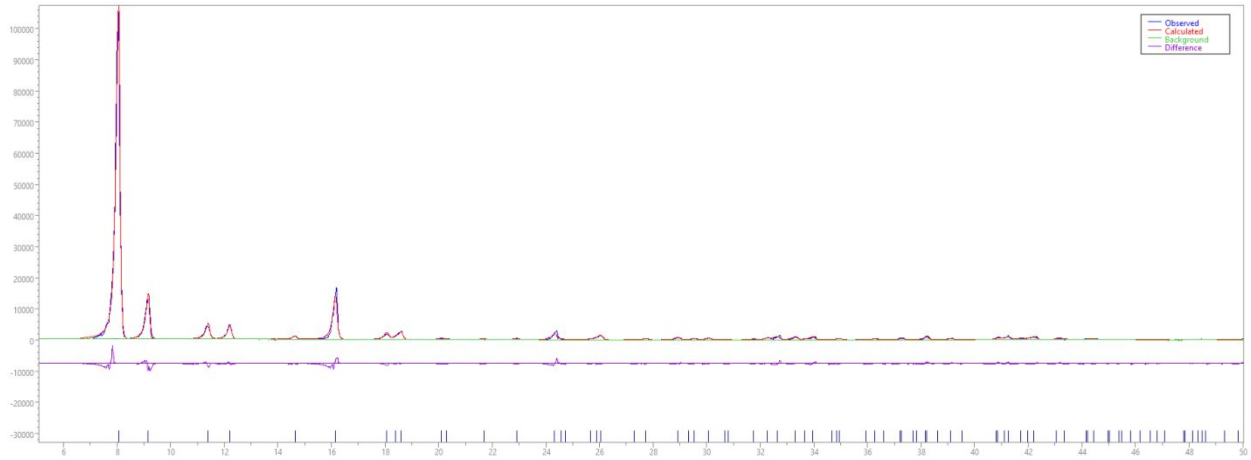

Figure S4a) Le Bail fit of guest-free **1**. Indexing was carried out by N-TREOR09 on EXPO2014. The crystal system was found to be tetragonal. The lattice parameters were refined to be $a = b = 10.98$ Å, $c = 9.654$ Å, $\alpha=\beta=\gamma= 90^{\circ}$, V = 1162.6 Å^3. The space group was found to be *P*4/*mmm*. General formula $Zn_2C_{18}H_{16}N_4O_8$. The reliability (*R*) factor based on the powder profile *Rp* was 7.51%. y axis (counts), x axis (2theta).

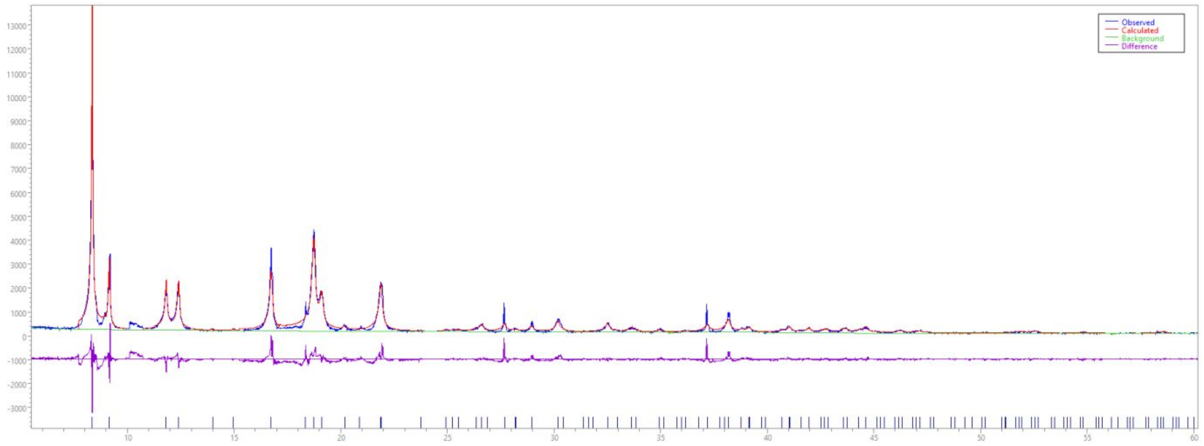

S4b) Le Bail fit of **1**$\supset$AP. Indexing was carried out by N-TREOR09 on EXPO2014. The crystal system was found to be tetragonal. The lattice parameters were refined to be $a = b = 15.06$ Å, $c = 19.35$ Å, $\alpha=\beta=\gamma= 90^{\circ}$, V = 4389.9 Å^3. The space group was found to be *I*4/*mcm*. General formula $Zn_8C_{122}H_{114}N_{36}O_{32}$. The reliability (*R*) factor based on the powder profile *Rp* was 6.23%. y axis (counts), x axis (2theta).

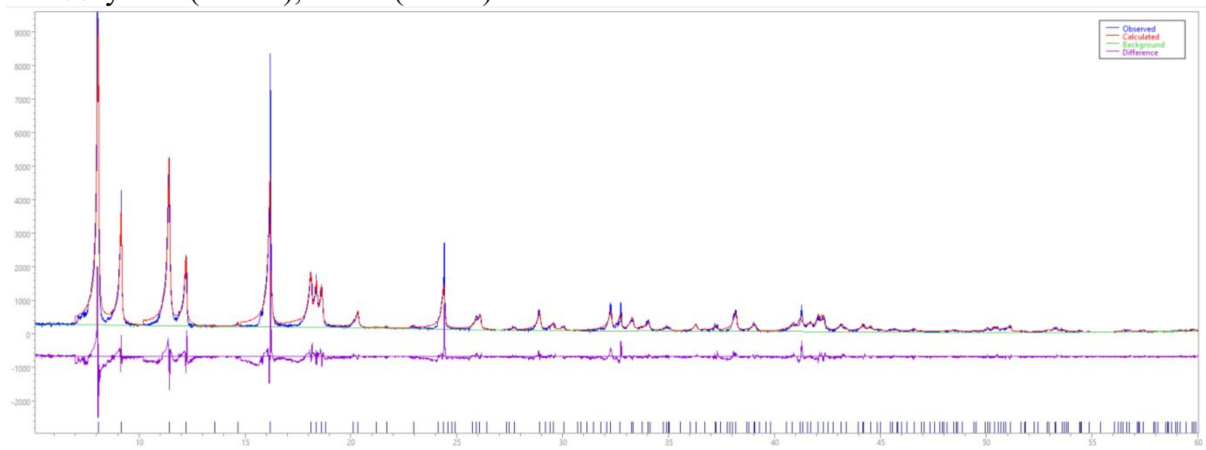

S4c) Le Bail fit of **1**⊃MOAP. Indexing was carried out by N-TREOR09 on EXPO2014. The crystal system was found to be tetragonal. The lattice parameters were refined to be $a = b =$ 15.41 Å, c = 19.31 Å, $\alpha=\beta=\gamma$ = 90˚, V = 4583.8 Å^3. The space group was found to be *I*4/*mcm*. General formula $Zn_8C_{116}H_{112}N_{32}O_{36}$. The reliability (*R*) factor based on the powder profile *Rp* was 7.73%. y axis (counts), x axis (2theta).

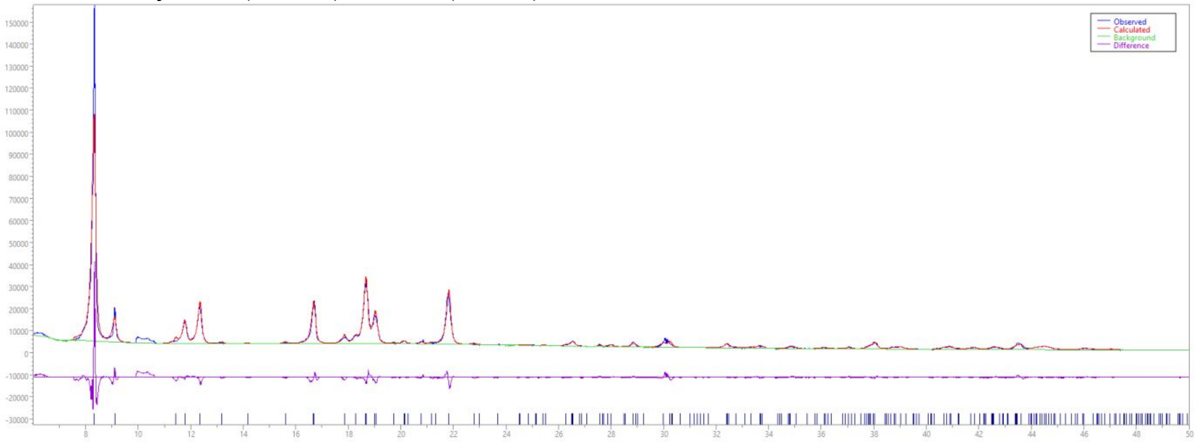

S4d) Le Bail fit of **1**⊃F-AP. Indexing was carried out by N-TREOR09 on EXPO2014. The crystal system was found to be tetragonal. The lattice parameters were refined to be $a = b =$ 15.05 Å, c = 19.35 Å, $\alpha=\beta=\gamma$ = 90˚, V = 4383.0 Å^3. The space group was found to be *I*4/*mcm*. General formula $Zn_8C_{122}H_{104}N_{36}O_{32}F_{10}$. The reliability (*R*) factor based on the powder profile *Rp* was 5.55%. y axis (counts), x axis (2theta).

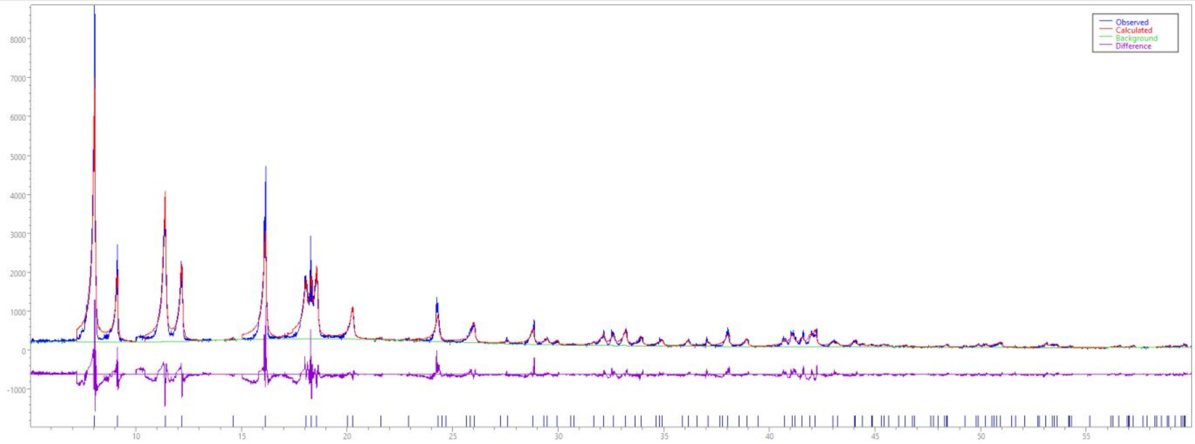

S4e) Le Bail fit of **1**⊃F-MOAP. Indexing was carried out by N-TREOR09 on EXPO2014. The crystal system was found to be tetragonal. The lattice parameters were refined to be $a = b =$ 15.38 Å, c = 19.31 Å, $\alpha=\beta=\gamma$ = 90˚, V = 4570.5 Å^3. The space group was found to be *I*4/*mcm*. General formula $Zn_8C_{116}H_{104}N_{32}O_{36}F_8$. The reliability (*R*) factor based on the powder profile *Rp* was 8.97%. y axis (counts), x axis (2theta).

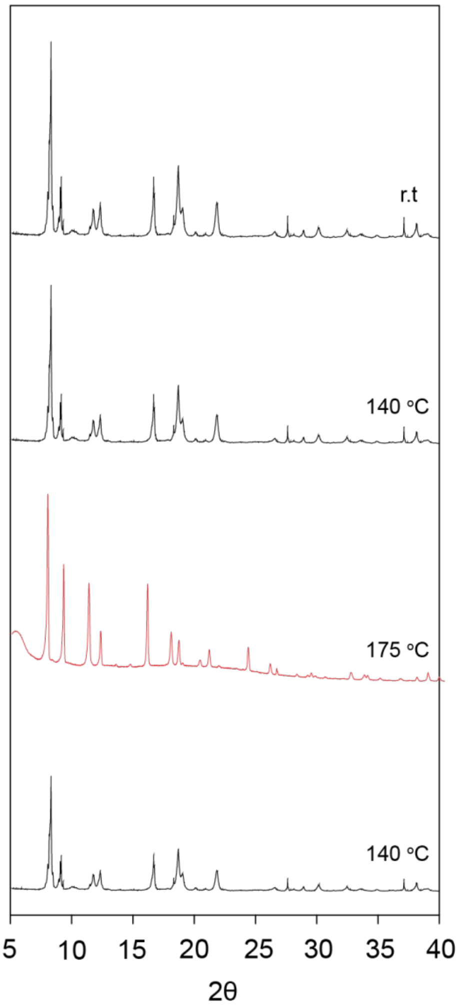

Figure S4f. XRPD profiles for synthesised **1**⊃AP, **1**⊃F-AP, **1**⊃MOAP, and **1**⊃F-MOAP. Through the occlusion of guest molecules, the framework structure remains stable in air and no changes are seen in the XRPD pattern over a period of 1 week.

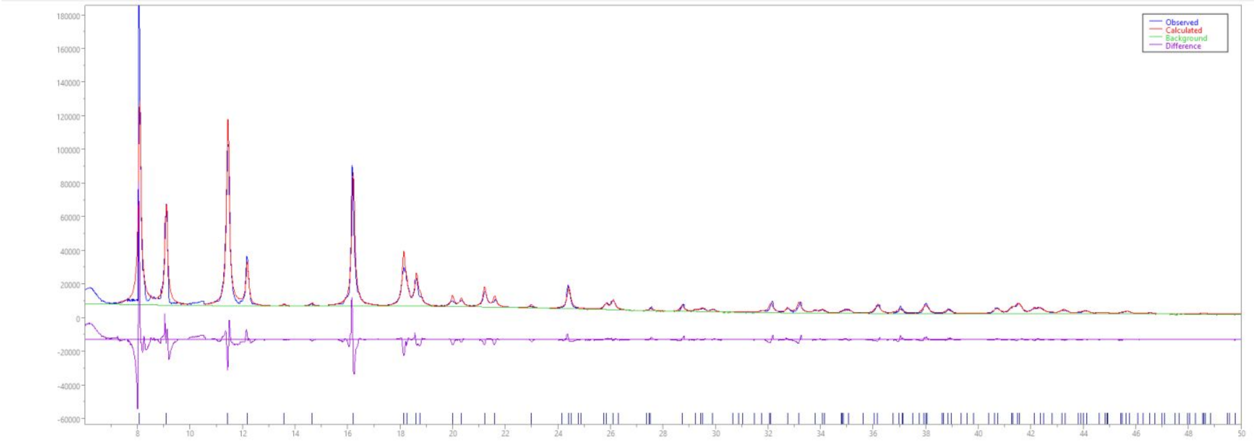

Figure S4g) Le Bail fit of **1**⊃AP at 180 °C. Indexing was carried out by N-TREOR09 on EXPO2014. The crystal system was found to be tetragonal. The lattice parameters were refined to be $a = b = 10.96$ Å, $c = 9.69$ Å, $\alpha=\beta=\gamma= 90^{\circ}$, V = 1166.9 Å^3. The space group was found to be *P*4/*mmm*. General formula $Zn_8C_{122}H_{114}N_{36}O_{32}$. The reliability (*R*) factor based on the powder profile *Rp* was 8.45%. y axis (counts), x axis (2theta).

Table S1: Crystallographic Details for **1**⊃AAPs.

Compound	**1**⊃AP	**1**⊃MOAP	**1**⊃F-AP	**1**⊃F-MOAP	**1**⊃AP (180 °C)
Empirical formula	$Zn_2C_{18}H_{16}N_4O_8$•1.25($C_{10}H_{10}N_4$)	$Zn_2C_{18}H_{16}N_4O_8$•($C_{11}H_{12}N_4O_1$)	$Zn_2C_{18}H_{16}N_4O_8$•1.25($C_{10}H_8N_4F_2$)	$Zn_2C_{18}H_{16}N_4O_8$•($C_{11}H_{10}N_4O_1F_2$)	$Zn_2C_{18}H_{16}N_4O_8$•1.25($C_{10}H_{10}N_4$)
Formula weight	779.88	763.35	824.85	799.33	779.88
Crystal system	tetragonal	tetragonal	tetragonal	tetragonal	tetragonal
Space group	*I*4/*mcm*	*I*4/*mcm*	*I*4/*mcm*	*I*4/*mcm*	*P*4/*mmm*
a/Å	15.06	15.41	15.05	15.38	10.96
b/Å	15.06	15.41	15.05	15.38	10.96
c/Å	19.35	19.31	19.35	19.31	9.69
α/°	90	90	90	90	90
β/°	90	90	90	90	90
γ/°	90	90	90	90	90
Volume/ $Å^3$	4389.9	4583.8	4383.0	4570.5	1166.9
Z	4	4	4	4	1
Radiation λ (Å)	1.5406	1.5406	1.5406	1.5406	1.5406
2Θ range for data collection /°	5-60	5-60	5-60	5-60	5-60
R_p	0.0623	0.0773	0.0555	0.0897	0.0897

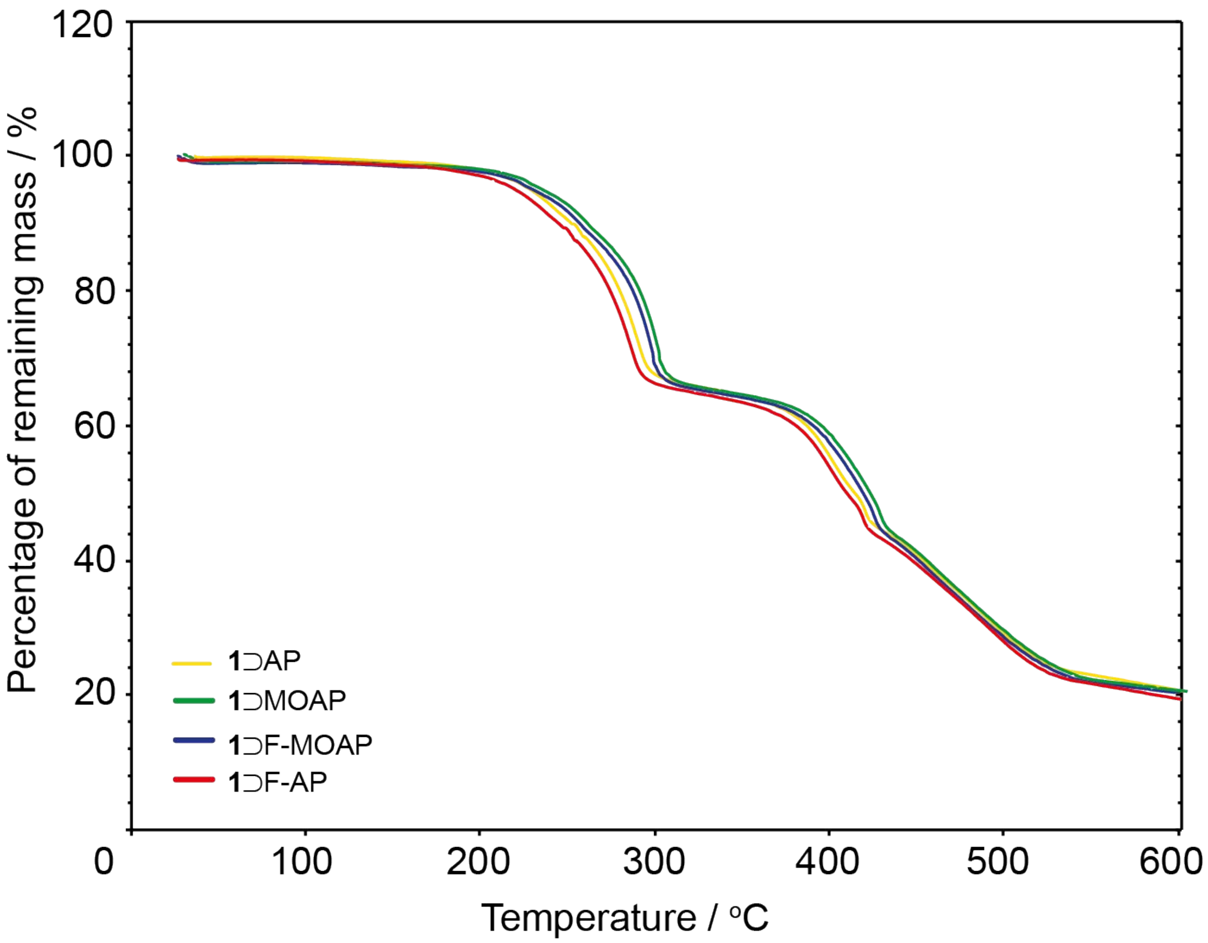

Figure S5. TGA profiles of **1**⊃AAPs. The expected remaining mass based on thermal decomposition to ZnO is; **1**⊃AP, 20.1%; **1**⊃MOAP, 20.5%; **1**⊃F-AP, 19.0%; **1**⊃F-MOAP, 19.6%. Experimental values range between 19.0 – 20.5%.

Single molecule calculations for each guest molecule isomer, confined in a 20 x 20 x 20 Angstrom box, were calculated to determine the expected resonance. These are shown below and labelled ‘δ_{iso} and ‘’δ_{iso}. To simulate the effects of fast rotational dynamics of the rings around the C-N bonds, chemical shifts were averaged for each carbon site across the two conformations, δ_{iso}^{av}.

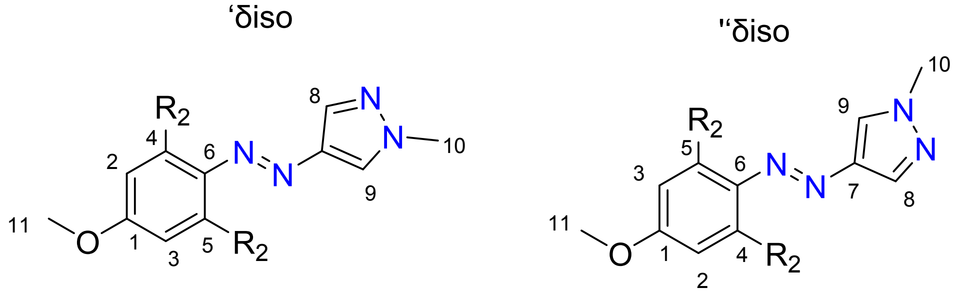

Table S2a. DFT calculated chemical shifts for *E*-AAPs.

C site	‘δ_{iso} (ppm)	‘’δ_{iso} (ppm)	δ_{iso}^{av} (ppm)
1	128.3	128.5	128.4
2	126.8	126.8	126.8
3	126.7	126.9	126.8
4	132.9	110.0	121.4
5	109.7	132.5	121.1
6	153.5	153.8	153.6
7	145.9	147.5	146.7
8	124.8	140.6	132.7
9	130.4	109.2	119.8
10	32.2	32.6	32.4

Table S2b. DFT calculated chemical shifts for *E*-MOAP.

C site	‘δ_{iso} (ppm)	‘’δ_{iso} (ppm)	δ_{iso}^{av} (ppm)
1	162.5	166.9	164.7
2	104.2	121.3	112.7
3	115.6	120.7	118.2
4	134.1	111.6	122.9
5	111.6	133.9	122.8
6	147.9	150.5	149.2
7	145.8	146.4	146.1
8	125.0	141.7	133.3
9	128.8	109.7	119.3
10	31.8	32.6	32.2
11	49.6	61.3	55.5

Table S2c. DFT calculated chemical shifts for *E*-F-AP.

C site	‘δ_{iso} (ppm)	‘’δ_{iso} (ppm)	δ_{iso}^{av} (ppm)
1	126.4	126.6	126.5
2	108.2	110.1	109.1
3	109.7	108.3	109.0
4	167.1	157.7	162.4
5	157.5	167.1	162.3
6	132.2	132.3	132.2
7	147.9	149.2	148.6
8	123.6	142.2	132.9
9	131.8	107.6	119.7
10	32.1	32.6	32.3

Table S2d. DFT calculated chemical shifts for *E*-F-MOAP.

C site	‘δ_{iso} (ppm)	‘’δ_{iso} (ppm)	δ_{iso}^{av} (ppm)
1	160.4	160.3	160.4
2	88.6	89.9	89.2
3	98.1	96.3	97.2
4	167.7	157.3	162.5
5	158.8	166.7	162.7
6	126.9	127.1	127.0
7	147.3	147.1	147.2
8	123.6	142.7	133.2
9	130.5	109.8	120.2
10	31.8	32.5	32.2
11	49.9	50.2	50.0

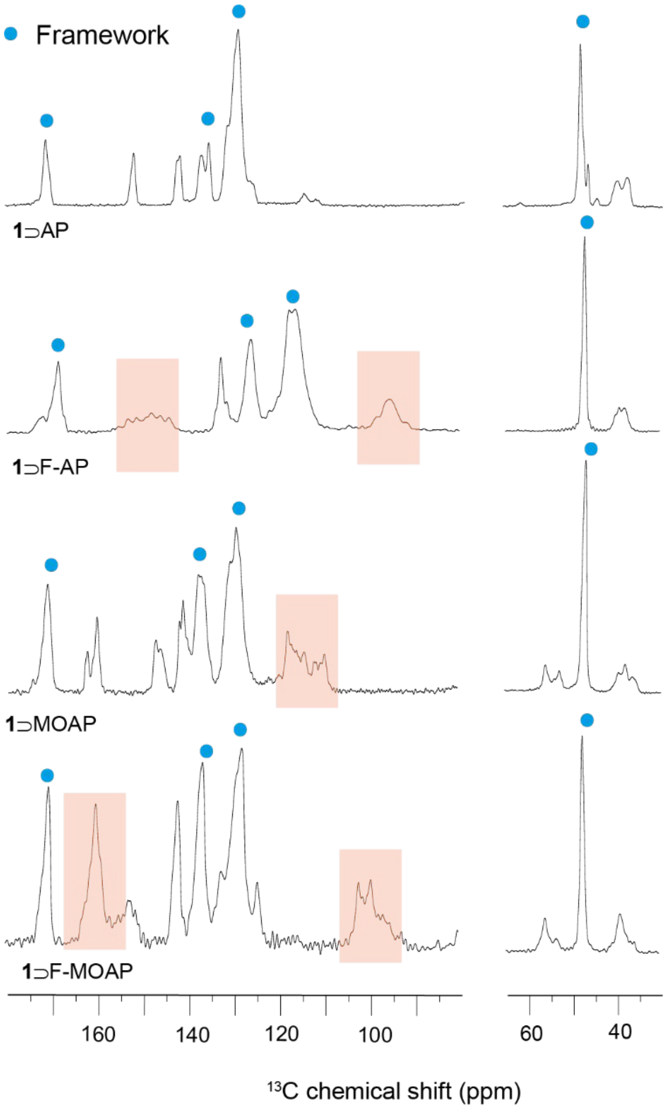

Figure S6. ^{13}C CPMAS NMR spectra of **1**⊃AAPs at 210 K. Framework resonances are indicated by blue dots. Guest resonances which show a shifting or broadening of their resonances from 310 K to 210 K are highlighted in orange.

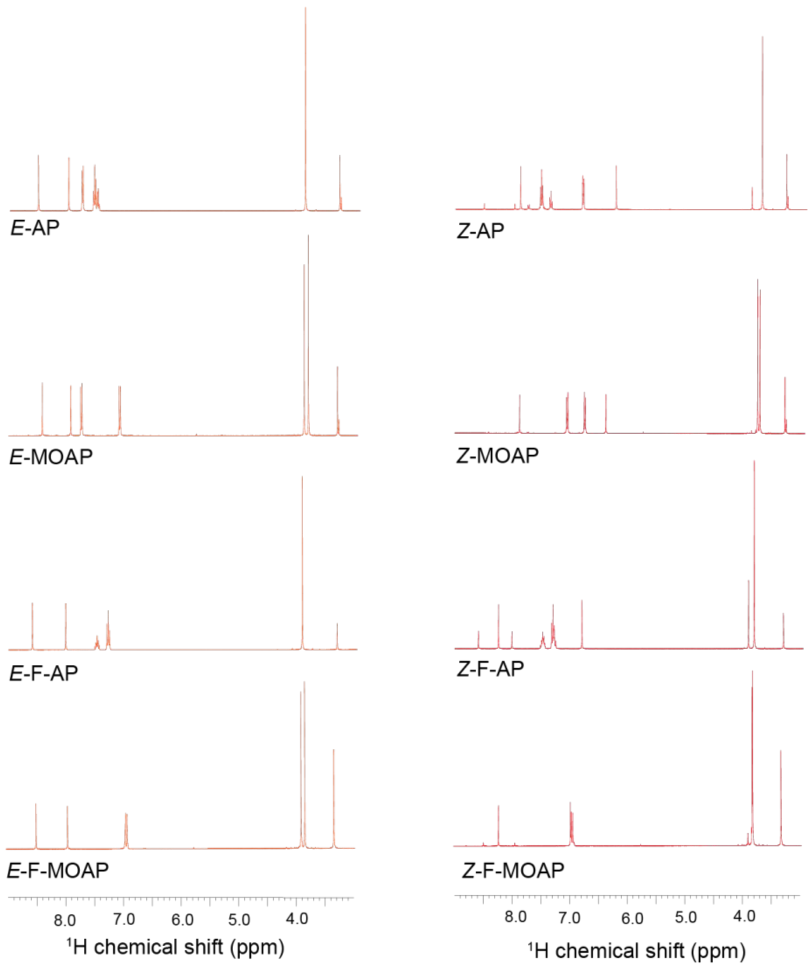

Figure S7. ^{1}H NMR spectra of guest molecules in deuterated benzene. *E*-isomers (left) and Z-isomer (right). A Bruker Avance III 400 NMR spectrometer with a 5 mm ^{1}H-X broadband observe probe was used to collect ^{1}H NMR data.

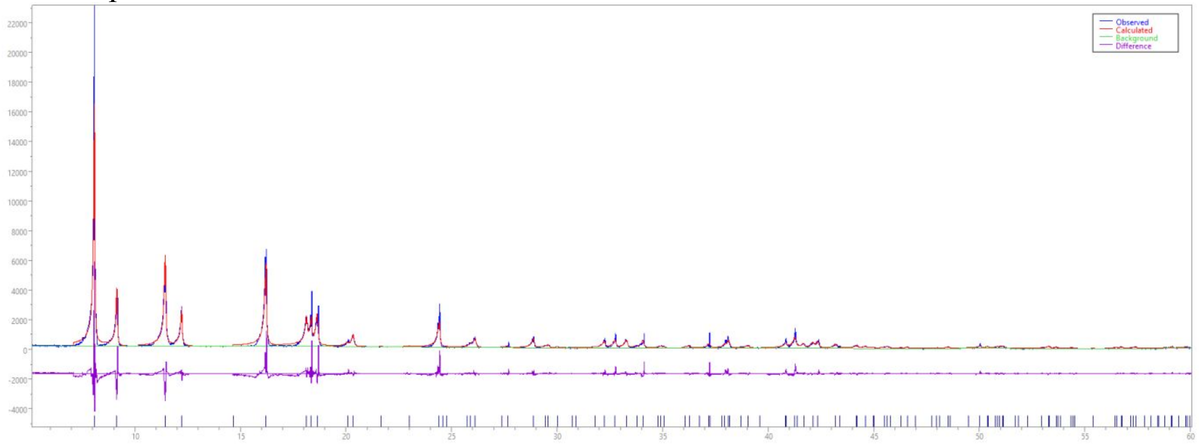

Figure S8a) Le Bail fit of irradiated **1**⊃AP. Indexing was carried out by N-TREOR09 on EXPO2014. The crystal system was found to be tetragonal. The lattice parameters were refined to be $a = b = 10.94$ Å, $c = 9.66$ Å, $\alpha=\beta=\gamma = 90^{\circ}$, V = 1157.1 Å^{3}. The space group was found to be *P*4/*mmm*. General formula $Zn_8C_{122}H_{114}N_{36}O_{32}$. The reliability (*R*) factor based on the powder profile *Rp* was 5.39%. y axis (counts), x axis (2theta).

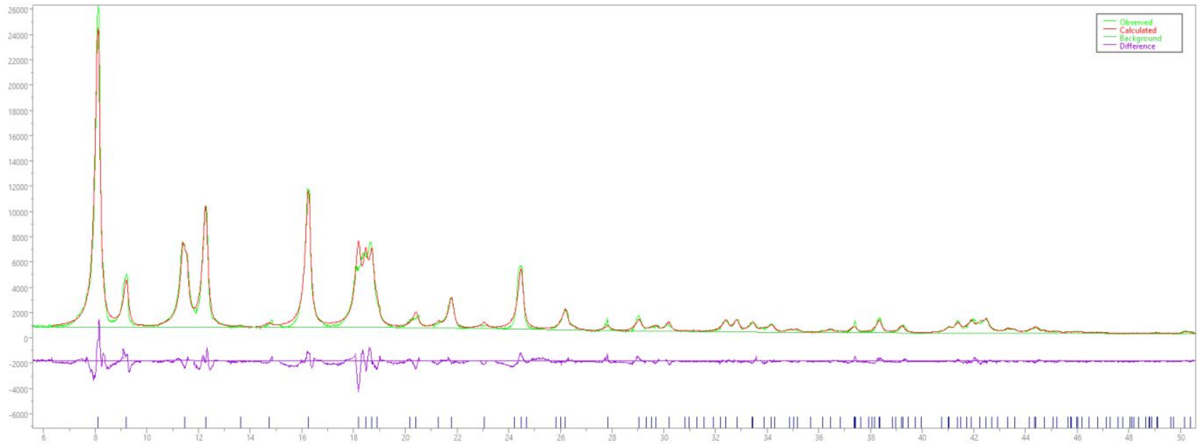

Figure S8b) Le Bail fit of irradiated **1**⊃F-AP. Indexing was carried out by N-TREOR09 on EXPO2014. The crystal system was found to be tetragonal. The lattice parameters were refined to be $a = b = 10.95$ Å, $c = 9.66$ Å, $\alpha=\beta=\gamma = 90^{\circ}$, V = 1157.6 Å^3. The space group was found to be *P*4/*mmm*. General formula $Zn_8C_{122}H_{104}N_{36}O_{32}F_{10}$. The reliability (*R*) factor based on the powder profile *Rp* was 3.60%. y axis (counts), x axis (2theta).

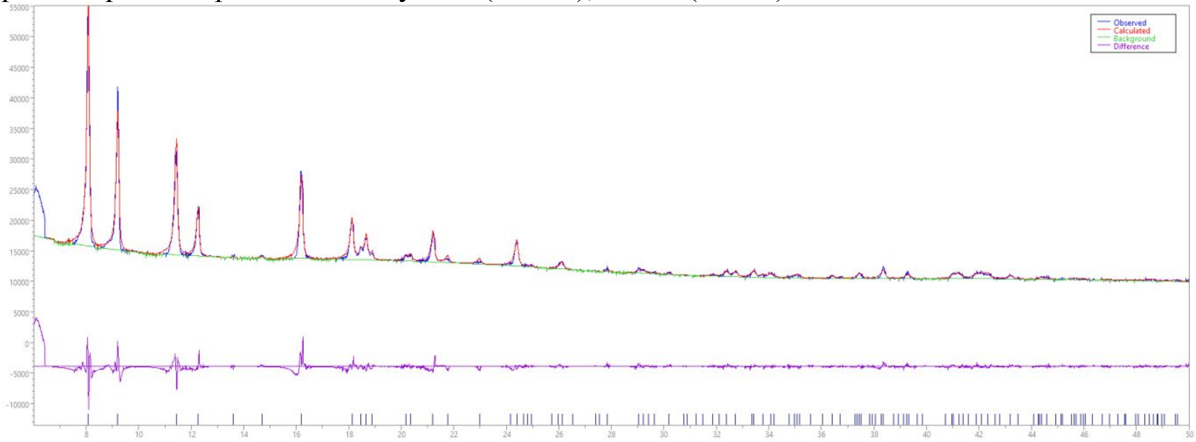

Figure S8c) Le Bail fit of irradiated **1**⊃MOAP. Indexing was carried out by N-TREOR09 on EXPO2014. The crystal system was found to be tetragonal. The lattice parameters were refined to be $a = b = 10.96$ Å, $c = 9.67$ Å, $\alpha=\beta=\gamma = 90^{\circ}$, V = 1161.2 Å^3. The space group was found to be *P*4/*mmm*. General formula $Zn_8C_{116}H_{112}N_{32}O_{36}$. The reliability (*R*) factor based on the powder profile *Rp* was 4.12%. y axis (counts), x axis (2theta).

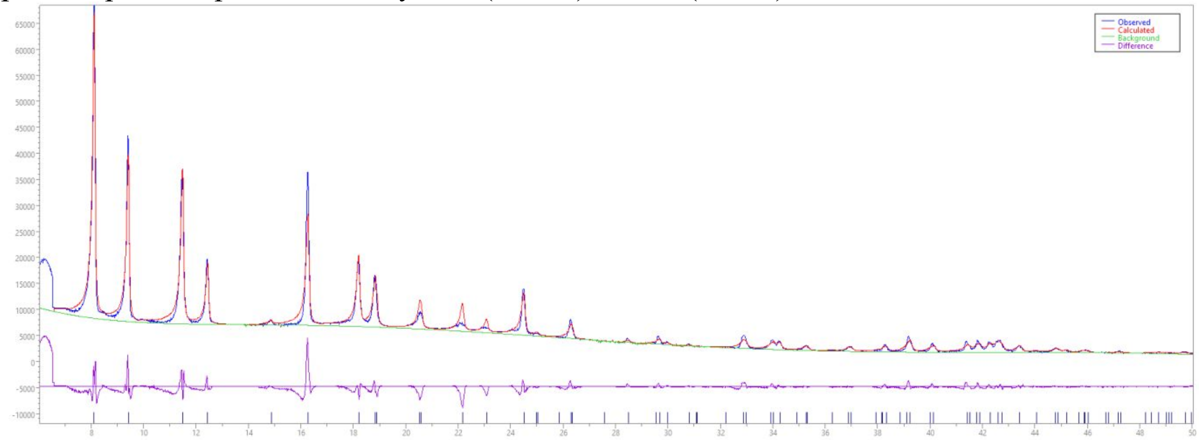

Figure S8d) Le Bail fit of irradiated **1**⊃F-MOAP. Indexing was carried out by N-TREOR09 on EXPO2014. The crystal system was found to be tetragonal. The lattice parameters were refined to be $a = b = 10.95$ Å, $c = 9.67$ Å, $\alpha=\beta=\gamma = 90^{\circ}$, V = 1156.9 Å^3. The space group was

found to be *P*4/*mmm*. General formula $Zn_8C_{116}H_{104}N_{32}O_{36}F_8$.. The reliability (*R*) factor based on the powder profile *Rp* was 6.69%. y axis (counts), x axis (2theta).

Table S3. Crystallographic Details for irradiated **1**⊃AAPs.

Compound	Irradiated **1**⊃AP	Irradiated **1**⊃MOAP	Irradiated **1**⊃F-AP	Irradiated **1**⊃F-MOAP
Empirical formula	$Zn_2C_{18}H_{16}N_4O_8$•1.25($C_{10}H_{10}N_4$)	$Zn_2C_{18}H_{16}N_4O_8$•($C_{11}H_{12}N_4O_1$)	$Zn_2C_{18}H_{16}N_4O_8$•1.25($C_{10}H_8N_4F_2$)	$Zn_2C_{18}H_{16}N_4O_8$•($C_{11}H_{10}N_4O_1F_2$)
Formula weight	779.88	763.35	824.85	799.33
Crystal system	tetragonal	tetragonal	tetragonal	tetragonal
Space group	*P*4/*mmm*	*P*4/*mmm*	*P*4/*mmm*	*P*4/*mmm*
a/Å	10.94	10.96	10.95	10.95
b/Å	10.94	10.96	10.95	10.95
c/Å	9.66	9.67	9.66	9.67
α/°	90	90	90	90
β/°	90	90	90	90
γ/°	90	90	90	90
Volume/Å^3	1157.1	1161.2	1157.6	1156.9
Z	1	1	1	1
Radiation λ (Å)	1.5406	1.5406	1.5406	1.5406
2Θ range for data collection/°	5-60	5-60	5-60	5-60
R_p	0.0539	0.0412	0.0360	0.0669

Table S4a. DFT calculated energy values for geometry optimised single molecules of AAPs and calculated energy difference between *E* and *Z* isomers.

	(energy/molecule) / (eV/molecule)	(energy/mole) / (eV/mol)	(energy/mol) / (J/mol)	(energy/g) / (J/g)	enthalpy / kJ mol^{-1}
E-AP	-2.8410 x 10^3	-1.7109 x 10^{27}	-2.7411 x 10^8	-1.5042 x 10^6	
Z-AP	-2.8405 x 10^3	-1.7106 x 10^{27}	-2.7406 x 10^8	-1.5040 x 10^6	
ED	-5.1513 x 10^1	-3.1022 x 10^{23}	- 5.7200 x 10^4	- 3.0720 x 10^2	- 57.2

Table S4b. DFT calculated energy values for geometry optimised single molecules of MOAP and calculated energy difference between *E* and *Z* isomers.

	(energy/molecule) / (eV/molecule)	(energy/mole) / (eV/mol)	(energy/mol) / (J/mol)	(energy/g) / (J/g)	enthalpy / kJ mol^{-1}
E-MOAP	-3.4701 x 10^3	-2.0897 x 10^{27}	-3.3481 x 10^8	-1.8373 x 10^6	
Z-MOAP	-3.4695 x 10^3	-2.0894 x 10^{27}	-3.3475 x 10^8	-1.8370 x 10^6	
ED	-5.8721 x 10^1	-3.5363 x 10^{23}	- 5.8700 x 10^4	- 2.7151 x 10^2	- 58.7

Table S4c. DFT calculated energy values for geometry optimised single molecules of F-AP and calculated energy difference between *E* and *Z* isomers.

	(energy/molecule) / (eV/molecule)	(energy/mole) / (eV/mol)	(energy/mol) / (J/mol)	(energy/g) / (J/g)	enthalpy / kJ mol^{-1}
E-F-AP	-4.1316 x 10^3	-2.4881 x 10^{27}	-3.9864 x 10^8	-2.1876 x 10^6	
Z-F-AP	-4.1313 x 10^3	-2.4879 x 10^{27}	-3.9861 x 10^8	-2.1875 x 10^6	
ED	-2.6450 x 10^1	-3.5929 x 10^{23}	- 5.6300 x 10^4	- 2.5338 x 10^2	- 56.3

Table S4d. DFT calculated energy values for geometry optimised single molecules of F-MOAP and calculated energy difference between *E* and *Z* isomers.

	(energy/molecule) / (eV/molecule)	(energy/mole) / (eV/mol)	(energy/mol) / (J/mol)	(energy/g) / (J/g)	enthalpy / kJ mol^{-1}
E-F-MOAP	-4.7607×10^{3}	-2.8669×10^{27}	-4.5933×10^{8}	-2.5207×10^{6}	
Z-F-MOAP	-4.7602×10^{3}	-2.8667×10^{27}	-4.5929×10^{8}	-2.5205×10^{6}	
ED	-4.1727×10^{1}	-2.5128×10^{23}	4.7600×10^{4}	1.8874×10^{2}	47.6

Table S5. Predicted energy differences on the heating branch of irradiated samples due to Z – E thermal relaxation v calculated values.

	mass of molecule	RMM	fractional mass of AP	Calculated energy at Z = 100% / J g^{-1}	PSS	Calculated energy at Z = PSS % / J g^{-1}	Experimental energy difference on the heating branch / J g^{-1}	Difference between calculated v Experimental / J g^{-1}
**1**	579.25	-	-	-	-	-	-	-
**1**-AP	186.2	812.0	0.29	88.1	0.57	50.2	-50.2	0.0
**1**-FAP	222.2	857.0	0.30	76.0	0.48	34.2	-30.7	3.5
**1**-MOAP	216.2	795.5	0.27	73.8	0.26	19.2	-18.3	0.9
**1**-F-MOAP	252.2	831.5	0.30	57.2	0.19	10.9	-10.5	0.4

Gas sorption analysis

Surface areas for the three samples obtained from BET analysis of the isotherms are summarised in **Table S8**. Samples **1**⊃AP and **1**⊃AP (irradiated) showed negligible adsorption which is attributed to the presence of the guest molecules within the pores preventing gas uptake. For guest-free **1**, a surface area of 312 ±8 m^2 g^{-1} is calculated. This is significantly lower than the literature value of 1450 m^2 g^{-1} (Dybtsev *et al*., *Angew. Chem. - Int. Ed.* 2004, 43, 5033); we attribute this to exposure to air during sample transfer to the BET analyser, which is known to cause decomposition of the structure. We note that samples of **1** loaded with AAP guest molecules remain stable in air for many weeks, further confirming that the presence of guest molecules inside the pores prevents gas molecules (atmospheric H_2O vapour) from entering the pores to cause decomposition of the framework.

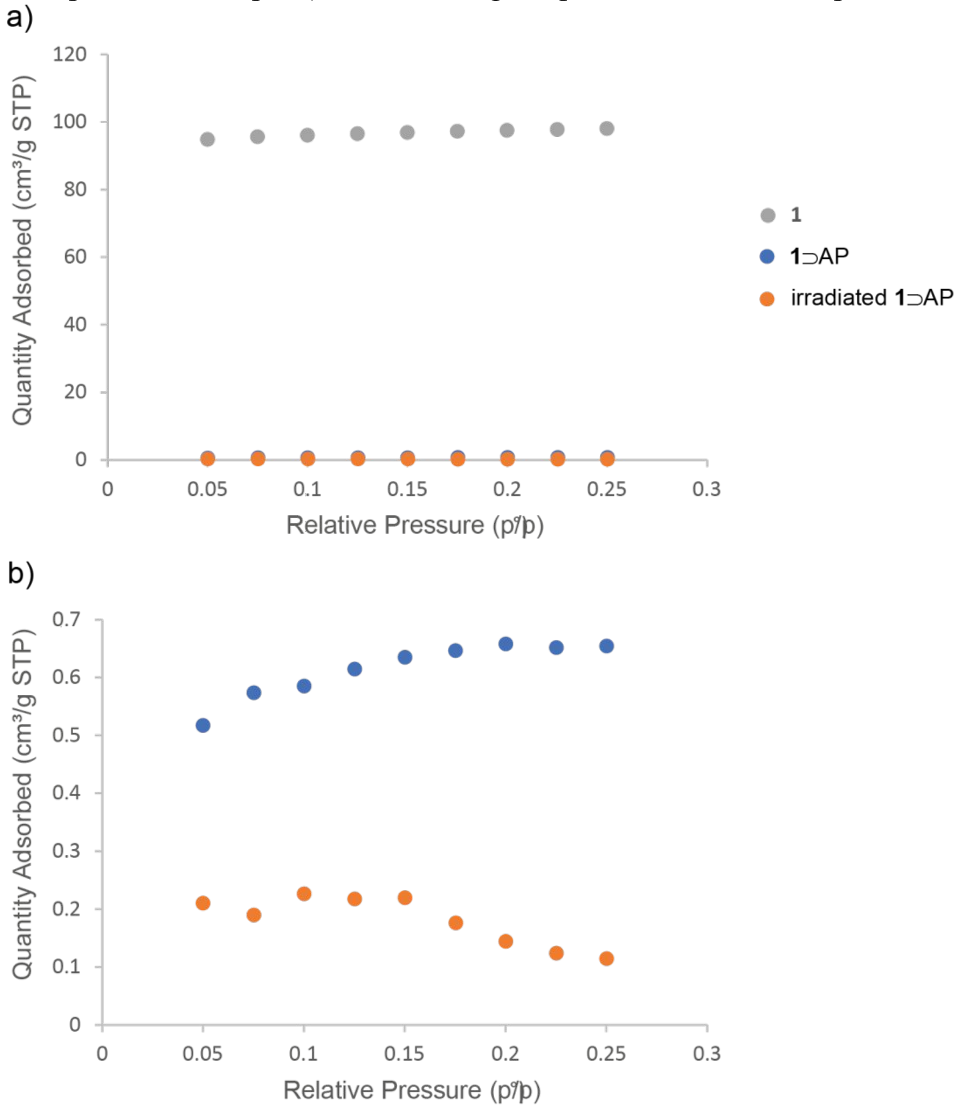

Figure S9 (a) N_2 gas adsorption isotherm for **1, 1**⊃AP, and **1**⊃AP irradiated for 5 hours under 365 nm light. (b) N_2 gas adsorption isotherm for **1**⊃AP and irradiated **1**⊃AP. Isotherms were measured using a Micromeritics gas sorption analyser.

Table S6. BET surface areas for samples studied by N_2 gas sorption

Sample	BET surface area / m^2 g^{-1}
Guest-free **1**	312 ± 8
**1**⊃AP	2.18 ± 0.07
**1**⊃AP irradiated 5 hours under 365 nm light	0.36 ± 0.05

DFT Calculation of ^{13}C chemical shifts for guest molecules

Single molecule calculations for each guest molecule isomer, confined in a 20 x 20 x 20 Angstrom box, were calculated to determine the expected resonance. These are shown below and labelled 'δ_{iso} and ''δ_{iso}. The equivalent number carbons were averaged to determine the chemical shift for a dynamic guest molecule undergoing ring flipping within the MOF pores, δ_{iso}.

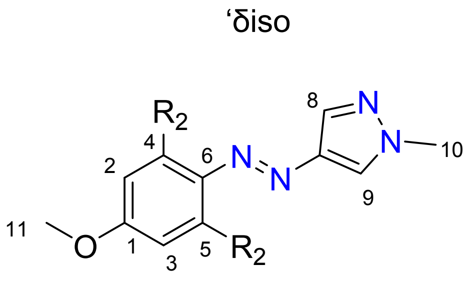

Table S7a. DFT calculated chemical shifts for *Z*-AAPs.

C site	'δ_{iso} (ppm)	''δ_{iso} (ppm)	δ_{iso} (ppm)
1	121.8	122.1	121.9
2	128.6	127.9	128.2
3	128.5	128.2	128.4
4	111.4	110.3	110.8
5	111.3	114.6	112.9
6	168.1	166.1	167.1
7	139.4	143.0	141.2
8	147.4	135.2	141.3
9	114.9	125.6	120.2
10	32.0	32.0	32.0

Table S7b. DFT calculated chemical shifts for *Z*-MOAP.

C site	'δ_{iso} (ppm)	''δ_{iso} (ppm)	δ_{iso} (ppm)
1	158.7	158.9	158.8
2	106.4	106.1	106.2
3	115.3	115.0	115.1
4	118.2	120.0	119.1
5	111.3	112.1	111.7
6	156.1	155.0	155.5
7	138.3	140.8	139.5
8	147.1	135.0	141.1
9	112.8	125.6	119.2
10	31.9	31.9	31.9
11	49.5	49.5	49.5

Table S7c. DFT calculated chemical shifts for *Z*-F-AP.

C site	‘δ_{iso} (ppm)	‘’δ_{iso} (ppm)	δ_{iso} (ppm)
1	122.8	122.6	122.7
2	109.0	108.8	108.9
3	108.3	108.2	108.3
4	160.5	159.0	159.8
5	148.2	150.1	149.2
6	137.4	138.8	138.1
7	142.8	143.2	143.0
8	145.5	136.5	141.0
9	110.1	125.2	117.6
10	32.6	32.2	32.4

Table S7d. DFT calculated chemical shifts for *Z*-F-MOAP.

C site	‘δ_{iso} (ppm)	‘’δ_{iso} (ppm)	δ_{iso} (ppm)
1	159.0	159.1	159.1
2	89.7	89.4	89.6
3	96.2	96.2	96.2
4	160.5	160.9	160.7
5	150.5	151.0	150.7
6	130.6	130.8	130.7
7	141.6	143.6	142.6
8	147.4	136.1	141.8
9	111.1	123.9	117.5
10	32.3	32.1	32.2
11	50.1	50.1	50.1

Table S8a. Change in the *Z*-isomer population in irradiated **1**⊃AP over 70 days.

Time / days	Z-isomer / %	N_t/N_0	$Ln(N_t/N_0)$
0	57.2	1.000	0.000
1	57.1	0.998	-0.002
7	56.9	0.995	-0.005
14	56.7	0.991	-0.009
21	56.5	0.988	-0.012
28	56.4	0.986	-0.014
35	56.0	0.979	-0.021
42	55.9	0.978	-0.023
49	55.7	0.974	-0.026
56	55.5	0.971	-0.030
63	55.3	0.967	-0.033
70	55.1	0.964	-0.037

Table S8b. Change in the *Z*-isomer population in irradiated **1**⊃F-AP over 70 days.

Time / days	Z-isomer / %	N_t/N_0	$Ln(N_t/N_0)$
0	50.0	1.000	0.000
1	49.9	0.998	-0.002
7	49.9	0.998	-0.002
14	49.9	0.998	-0.002
21	49.8	0.996	-0.004
28	49.8	0.996	-0.004
35	49.8	0.996	-0.004
42	49.7	0.994	-0.006
49	49.7	0.994	-0.006
56	49.7	0.994	-0.006
63	49.6	0.992	-0.008
70	49.6	0.992	-0.008

Table S8c. Change in the *Z*-isomer population in irradiated **1**⊃MOAP over 70 days.

Time / days	Z-isomer / %	N_t/N_0	$Ln(N_t/N_0)$
0	26.0	1.000	0.000
1	26.0	1.000	0.000
7	25.8	0.992	-0.008
14	25.5	0.981	-0.019
21	25.4	0.977	-0.023
28	25.2	0.969	-0.031
35	25.0	0.962	-0.039
42	24.8	0.954	-0.047
49	24.6	0.946	-0.055
56	24.3	0.935	-0.068
63	24.2	0.931	-0.072
70	24.0	0.923	-0.080

Table S8d. Change in the *Z*-isomer population in irradiated **1**⊃F-MOAP over 70 days.

Time / days	Z-isomer / %	N_t/N_0	$Ln(N_t/N_0)$
0	19.4	1.000	0.000
1	19.4	1.000	0.000
7	19.4	1.000	0.000
14	19.4	1.000	0.000
21	19.4	1.000	0.000
28	19.4	1.000	0.000
35	19.3	0.995	-0.005
42	19.3	0.995	-0.005
49	19.3	0.995	-0.005
56	19.3	0.995	-0.005
63	19.2	0.990	-0.010
70	19.2	0.990	-0.010

Assuming the metastable *cis* isomer undergoes thermal reconversion to the ground-state *E* isomer according to first-order kinetics, the number of guest molecules in the *Z* isomeric state as a function of time ($N_{cis}(t)$) can be expressed by

$$N_Z(t) = N_Z(0)e^{-\lambda t}$$

where $N_Z(0)$ is the number of guest molecules in the *Z* isomeric state at the beginning of the measurement and λ is a decay constant. This rearranges to the following form whereby plotting $\ln(N_Z(t)/N_Z(0))$ vs t allows determination of λ from the gradient of the straight line of best fit.

$$ln\left(\frac{N_Z(t)}{N_Z(0)}\right) = -\lambda t$$

The half-life, $t_{½}$, then given by

$$t_{\frac{1}{2}} = \frac{ln2}{\lambda}$$
